# Regulation of Synaptic *nlg-1*/Neuroligin Abundance by the *skn-1*/Nrf Stress Response Pathway Protects against Oxidative Stress

**DOI:** 10.1371/journal.pgen.1004100

**Published:** 2014-01-16

**Authors:** Trisha A. Staab, Oleg Egrafov, James A. Knowles, Derek Sieburth

**Affiliations:** 1 Integrative Biology of Disease Graduate Program, University of Southern California, Los Angeles, California, United States of America; 2 Zilkha Neurogenetic Institute, Keck School of Medicine, University of Southern California, Los Angeles, California, United States of America; University of California San Diego, United States of America

## Abstract

The Nrf family of transcription factors mediates adaptive responses to stress and longevity, but the identities of the crucial Nrf targets, and the tissues in which they function in multicellular organisms to promote survival, are not known. Here, we use whole transcriptome RNA sequencing to identify 810 genes whose expression is controlled by the SKN-1/Nrf2 negative regulator WDR-23 in the nervous system of *Caenorhabditis elegans*. Among the genes identified is the synaptic cell adhesion molecule *nlg-1/*neuroligin. We find that the synaptic abundance of NLG-1 protein increases following pharmacological treatments that generate oxidative stress or by the genetic activation of *skn-1*. Increasing *nlg-1* dosage correlates with increased survival in response to oxidative stress, whereas genetic inactivation of *nlg-1* reduces survival and impairs *skn-1-*mediated stress resistance. We identify a canonical SKN-1 binding site in the *nlg-1* promoter that binds to SKN-1 *in vitro* and is necessary for SKN-1 and toxin-mediated increases in *nlg-1* expression *in vivo*. Together, our results suggest that SKN-1 activation in the nervous system can confer protection to organisms in response to stress by directly regulating *nlg-1/*neuroligin expression.

## Author Summary

Organisms have evolved mechanisms to protect themselves at the cellular level in response to a variety of environmental stresses. Oxidative stress, caused by an imbalance in the cellular production of free radicals and endogenous antioxidant defenses, can be particularly detrimental to the nervous system. Indeed, elevated levels of oxidative stress have been linked to nearly all neurodegenerative diseases. Therefore, understanding how living creatures protect themselves against oxidative stress, from a cellular to a systemic level, is vital. Here, we have found that increased stress activates a pathway that increases the amount of certain proteins found at neuronal synapses. This presents an interesting model in which, in response to stress, neurons might attempt to enhance the strength of the synapse to prevent degeneration.

## Introduction

Oxidative stress is generated in cells when an imbalance occurs between the production of electrophilic reactive species and the endogenous defenses against these harmful molecules [1]. Increased reactive oxygen species (ROS) can cause damage to cellular components such as lipids, DNA, and proteins, and can result in a myriad of detrimental effects, including protein aggregation, changes in cell signaling, or altered cell cycle progression. Given the nervous system's high metabolic demands, high lipid and iron content, and low regenerative ability, it is not surprising that oxidative stress is particularly detrimental to this tissue; indeed, elevated levels of reactive species have been implicated in a range of neurodegenerative diseases including Parkinson's disease, Alzheimer's disease and amyotrophic lateral sclerosis [2]–[4]. In multicellular organisms, investigating pathways that can mitigate the consequences of elevated oxidative stress on a cellular and organismal level is an ongoing area of investigation.

The Nrf (nuclear factor E2-related factor) family of transcription factors controls the primary response to oxidative and xenobiotic stress in mammals [5]–[7]. Studies of Nrf2 have established a critical role for the transcription factor in defending tissues from oxidative damage [7], [8]. During normal conditions, Nrf2 is sequestered in the cytoplasm by the kelch-domain containing protein Keap1; in response to stress, Keap1 releases Nrf2, allowing the transcription factor to translocate into the nucleus and initiate transcription of downstream targets by binding to the antioxidant response element (ARE) in the promoter region of stress response genes [6], [9].

Nrf2 is ubiquitously expressed, and in neurons, increased Nrf2 activity protects against toxicity by hydrogen peroxide, glutamate, and mitochondrial toxins [10], [11]. Microarray studies using neuronal cultures lacking Nrf2 have identified detoxifying, antioxidant and defense genes to be regulated by the transcription factor [10]–[12]. Interestingly, these studies have also found changes in the expression of genes involved in a variety of processes, including cell signaling and calcium homeostasis, as well as neuron-specific genes, but the functional significance of these changes has not been determined.

In *C. elegans*, the Nrf family homolog SKN-1 [13] confers cellular and organismal protection from a variety of environmental stressors. Although most studies have examined detoxification and stress response in the intestine, a role for SKN-1 in the nervous system is emerging. SKN-1 functions in a pair of neurons to promote longevity [14], and systemic knockdown of *skn-1* increases dopaminergic neuron degeneration during methylmercury, aluminum and manganese toxicity [15]–[17]. Furthermore, loss of *skn-1* enhances manganese-induced organismal death [18]. In *C. elegans*, the abundance of SKN-1 is negatively regulated in part by the DDB-1/CUL-4 ubiquitin ligase substrate targeting protein WDR-23, which is proposed to function in an analogous manner as Keap1 to promote degradation of SKN-1 during non-stressed conditions. [19], [20]. Mutants lacking *wdr-23* have increased SKN-1 protein levels, express high levels of genes involved in antioxidant and xenobiotic responses, and are resistant to stress [19]–[21].

In this study, we find that SKN-1 is negatively regulated by WDR-23 in cholinergic motor neurons. Using whole transcriptome RNA sequencing (RNAseq) of *wdr-23* mutants expressing functional *wdr-23a* in the nervous system, we identify 810 genes whose expression is likely to be regulated by SKN-1; one of these is the cell adhesion molecule *nlg-1/*neuroligin. Both *nlg-1* expression in neurons and NLG-1 protein abundance at synapses increase in mutants with increased SKN-1 activity as well as in animals exposed to toxins that generate mitochondrial stress. Furthermore, increasing NLG-1 protein abundance enhances survival following toxin treatment, and loss of *nlg-1* diminishes SKN-1-mediated toxin resistance. Together, these results support a role for SKN-1 in promoting organismal survival by regulating synaptic neuroligin abundance.

## Results

### WDR-23 regulates SKN-1 abundance in neurons

**Figure 1:**
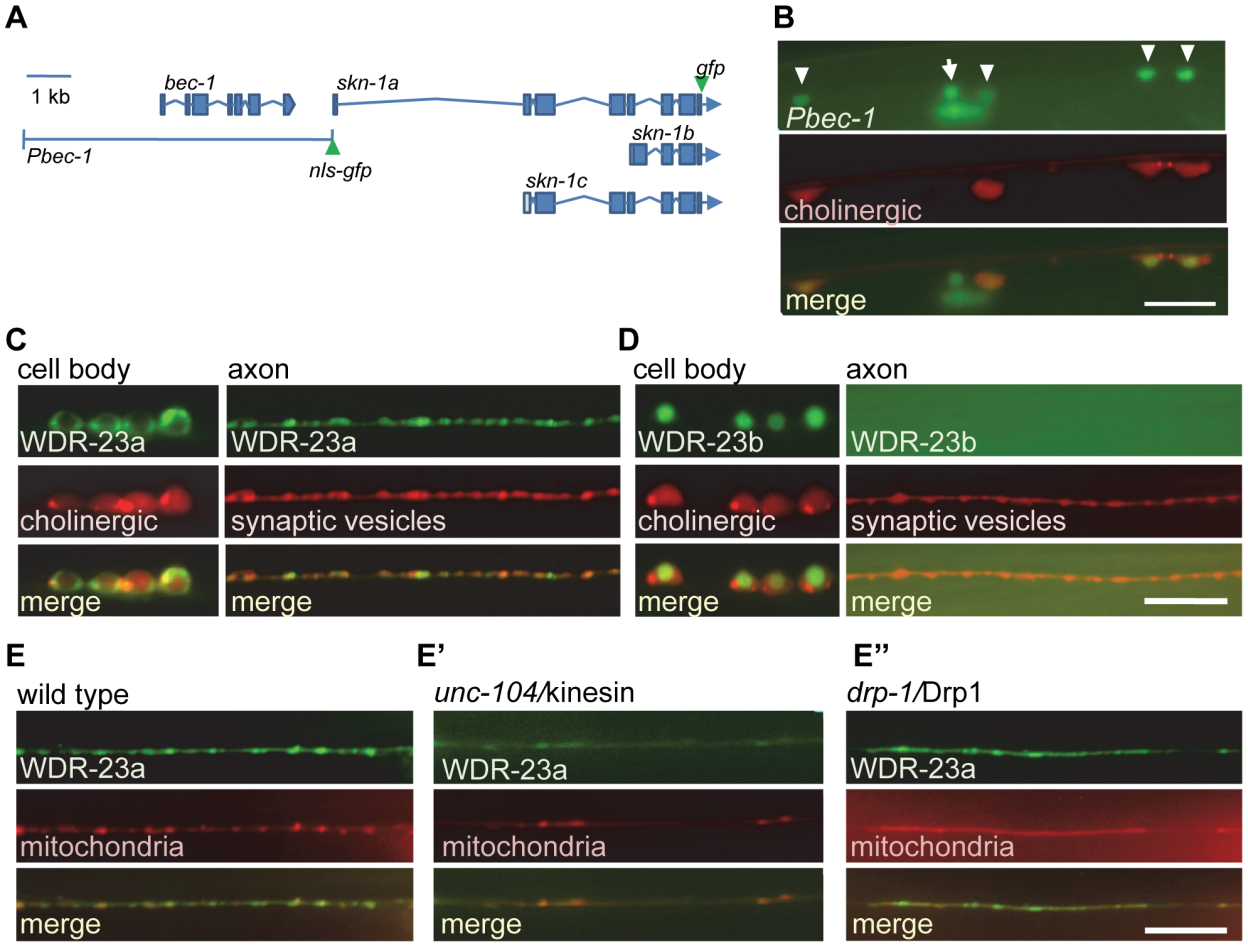
SKN-1 expression and WDR-23 sub-cellular localization in motor neurons. A) Schematic of *bec-1* and *skn-1* genetic loci. Locations of *gfp* tags used in this study indicated. B) Co-localization of *nls-gfp* driven by the 7.3 kb *Pbec-1* promoter (*vjEx254*) with *Punc17*::*rfp* (*nuIs321*) in cholinergic motor neurons. Arrowheads indicate cholinergic motor neurons; arrow indicates GABAergic neuron. C–D) *Left*, Localization of WDR-23a::GFP (*vjEx423*; C) and WDR-23b::GFP (*vjEx426*; D) in ventral cord motor neurons expressed under the *snb-1* promoter fragment. Neuronal cell bodies are identified by the *unc-17* promoter driving *rfp* (*nuIs321*) for reference. *Right*, WDR-23a::GFP (*vjIs26*; C) and WDR-23b::GFP (*vjEx7*; D) co-localization with the synaptic vesicle associated protein mCherry::SNB-1 (*vjEx339*) in motor neuron axons using the *unc-129* promoter fragment. E) WDR-23a::GFP (*vjIs26*) co-localization with the mitochondrial marker (INVOM::RFP; *vjEx663*) in motor neuron axons in wild type, *unc-104/*kinesin mutants (E′) and *drp-1/*Drp1 mutants (E″). Scale bars represent 10 µm.

The *skn-1* locus encodes three isoforms, *skn-1a*, *skn-1b* and *skn-1c* (wormbase.org), which differ in their N termini and utilize unique transcriptional start sites. *skn-1c* is primarily detected in the intestine; *skn-1b*, on the other hand, is expressed principally in a pair of sensory neurons and is involved in dietary restriction induced longevity [13], [14]. The expression of the longest isoform, *skn-1a*, however, has not been examined. To examine the expression of *skn-1a*, we generated a fluorescent reporter by using a 7.3 kb fragment upstream of the *skn-1a* start site to drive *gfp* fused to a nuclear localization signal (*nls-gfp*). *skn-1a* is a downstream gene within the *bec-1/*beclin operon [14], [22], [23], and the 7.3 kb fragment includes the *bec-1* coding region and 3.3 kb upstream of *bec-1* (Figure 1A). In animals expressing this construct, fluorescence was observed in many tissues, as previously reported [24], [25], as well as in cholinergic neurons of the ventral cord (labeled with the *unc-17*/VAChT promoter expressing mCherry) and GABAergic neurons (unlabeled ventral cord neurons; Figure 1B).

WDR-23 has two isoforms, WDR-23a and WDR-23b, which are expressed in neurons of the ventral cord [19]–[21]. In cholinergic neurons of the ventral cord, a WDR-23a::GFP fusion protein was found in cell bodies and in axons where it adopted a punctate pattern of fluorescence (Figure 1C), whereas WDR-23b::GFP localized exclusively to nuclei (Figure 1D). In axons, WDR-23a puncta co-localized with a synaptic vesicle marker (Figure 1C), and WDR-23a fluorescence became diffuse in animals lacking *unc-104/*kinesin (Figure 1E′), which is required for trafficking of organelles along microtubules in neurons [26]. This suggests that WDR-23a associates with presynaptic organelles. WDR-23a also co-localized with a mitochondrial marker at synapses (Figure 1E), and in *unc-104* mutants, WDR-23a puncta remained co-localized with displaced mitochondria in axons (Figure 1E′). In mutants defective in *drp-1/*Drp1, a protein required for normal mitochondrial fission [27], both WDR-23 and mitochondrial markers became displaced in axons (Figure 1E″). Together, these results suggest that WDR-23a may associate with presynaptic organelles, including mitochondria, at synapses. Consistent with this, WDR-23a co-localizes with outer mitochondrial membrane markers when expressed in muscle cells [20].

**Figure 2:**
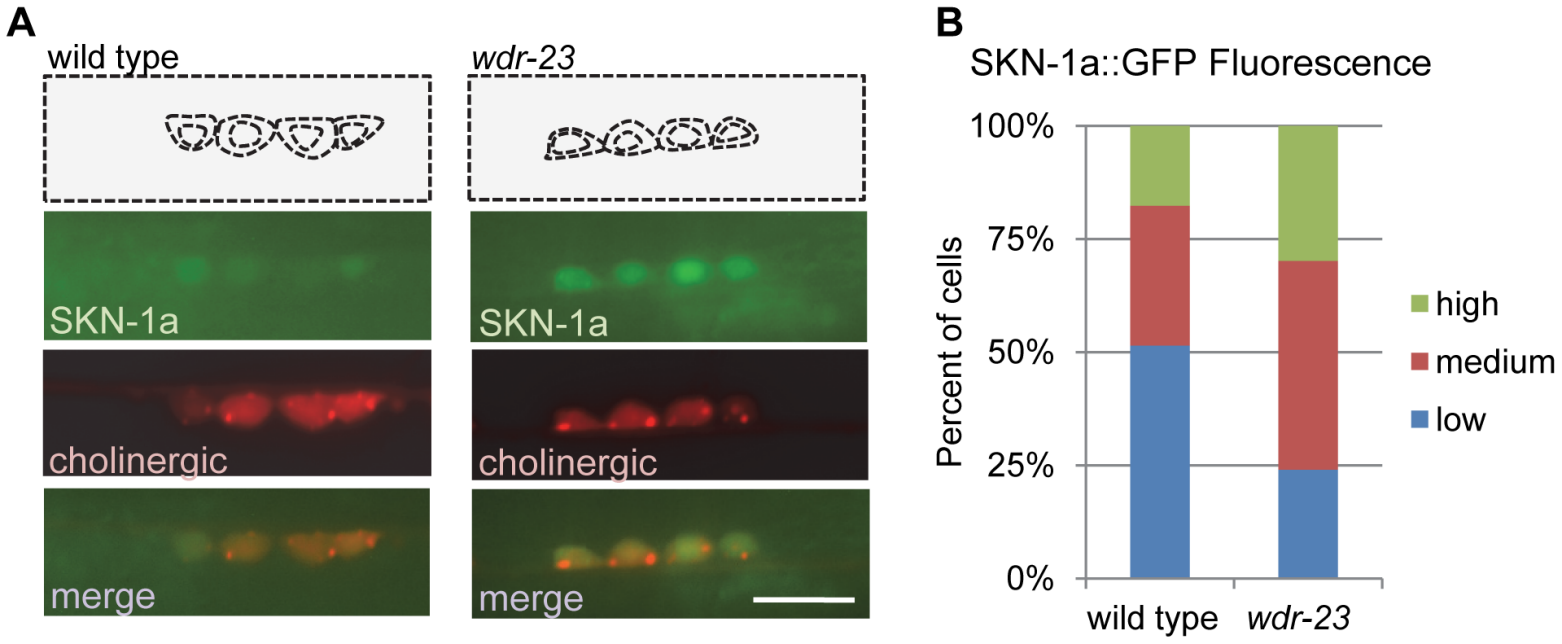
WDR-23 regulates SKN-1 abundance in motor neurons. A) Representative images of SKN-1a::GFP driven by the *unc-17* promoter (*vjIs45*) in cholinergic motor neurons of wild type, *left*, and *wdr-23* mutants, *right*. Cell bodies identified by presence of RFP expressed under the *unc-17* promoter (*nuIs321*). B) Quantification of SKN-1a::GFP (*vjIs45*) in wild type (N = 28) and *wdr-23* (N = 33) mutants. Cells were classified as low, medium or high expressing neurons based on average SKN-1a::GFP fluorescence per cell (see methods). Scale bar represents 10 µm.

To test whether WDR-23 regulates the abundance of SKN-1 in the nervous system, as it does in the intestine [19], [21], we examined the abundance of SKN-1a::GFP fusion proteins in motor neurons. We drove SKN-1a::GFP expression using the cholinergic *unc-17*/VAChT promoter since *unc-17* is not transcriptionally regulated by *wdr-23* [20], and therefore changes in SKN-1a::GFP should reflect changes in protein abundance and not *skn-1* expression. In wild type animals, SKN-1a::GFP fluorescence was detected in 49% (n = 272) of ventral cord cholinergic neurons (Figure 2A), consistent with low levels of SKN-1 reported in the intestine in non-stressed animals [13]. In *wdr-23* mutants, SKN-1a::GFP fluorescence was detected in a larger fraction of neurons compared to wild type animals (77%, n = 349). In addition, the average fluorescence intensity of SKN-1a::GFP in neuronal cell bodies of *wdr-23* mutants increased compared to wild type controls (Figures 2A and 2B; mean fluorescence±sem wt: 44.7±2.8; *wdr-23*: 62.6±3.1; *p*<0.001, Student's *t*-test). Together, these results suggest that WDR-23 negatively regulates the abundance of SKN-1a in motor neurons.

### A subset of neuronal genes is regulated by SKN-1

**Figure 3:**
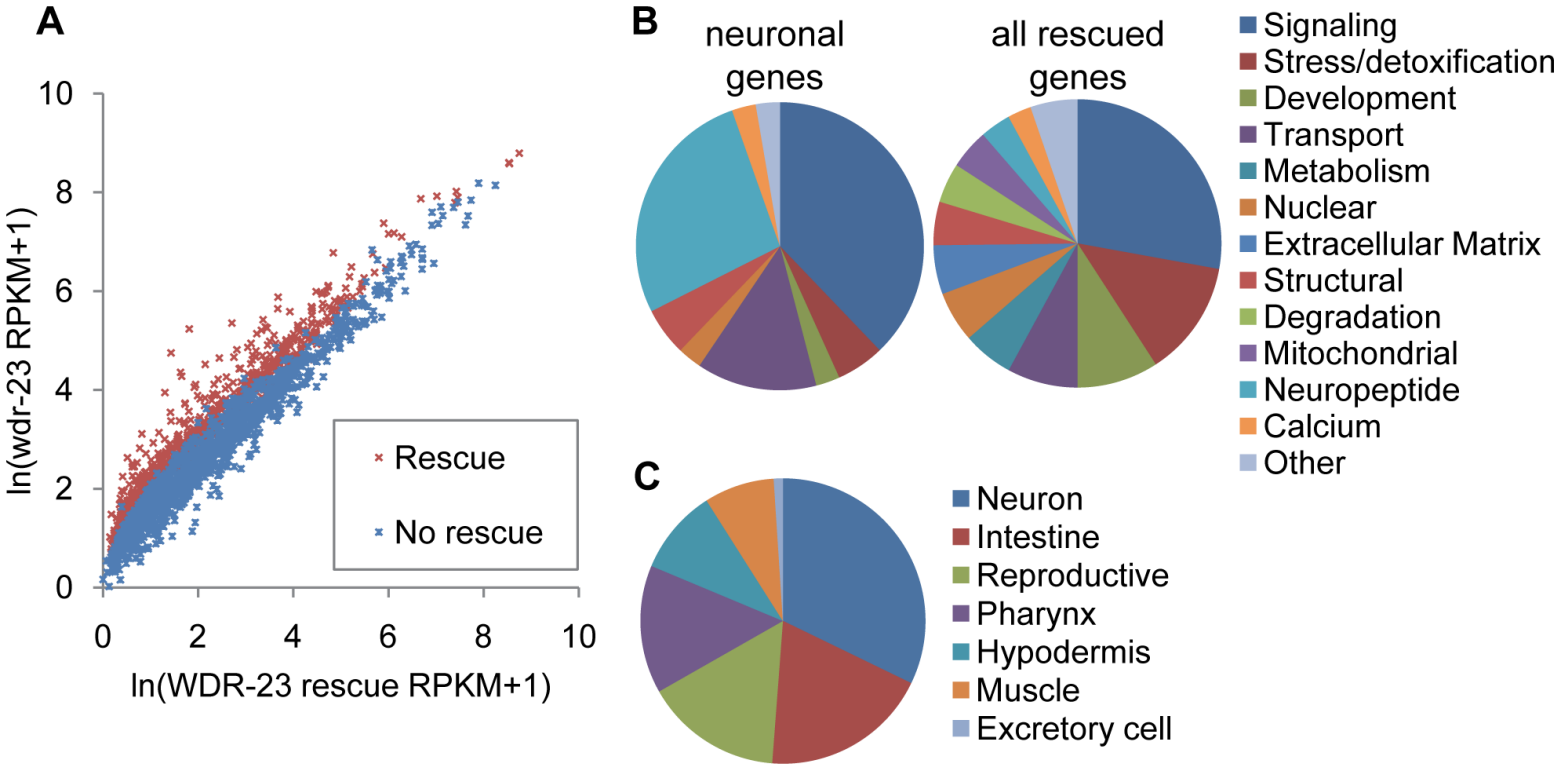
Analysis of whole transcriptome RNA sequencing of *wdr-23* mutants expressing neuronal WDR-23. A) Correlation plot of RNAseq reads per kilobase per million reads (RPKM) for WDR-23 rescue compared to *wdr-23* mutants, for 2,285 genes previously identified to be significantly different between wild type and *wdr-23* mutants [20]. Correlation plot includes 2,118 genes, as successful reads were not obtained for all genes in the rescuing line. WDR-23 rescue denotes *wdr-23* mutants with expression of WDR-23a (using *snb-1/*synaptobrevin promoter, *nuIs225*). Genes that do not rescue (indicated in blue) have similar RPKM for *wdr-23* mutants and the WDR-23 rescue, whereas genes that rescue (indicated in red) have statistically higher RPKM for *wdr-23* mutants. B-C) Characterization of 810 genes significantly different in *wdr-23* versus WDR-23 rescue (*nuIs225*). B) *Right*, Breakdown by GO term of genes with predicted functions identified by DAVID (492 genes). *Left*, Breakdown by GO term of rescued genes expressed specifically in the nervous system (43 genes). C) Distribution by tissue expression of genes with WormMart reported expression patterns (173 genes).

*wdr-23* mutants have a variety of developmental and behavioral defects including reduced locomotion, resistance to stress, small size, and developmental delay. Each of these defects is suppressed by loss of *skn-1* [19]–[21], suggesting that the primary function of WDR-23 is to negatively regulate SKN-1. We previously identified 2,285 transcripts that are significantly up-regulated in *wdr-23* mutants compared to wild type controls using RNAseq [20]. To identify genes among these that are regulated by WDR-23 specifically in the nervous system, we performed RNAseq of *wdr-23* mutants expressing an integrated array of full-length *wdr-23a* cDNA driven by the pan-neuronal *snb-1* promoter (referred to as WDR-23 rescue). Of the 2,285 genes whose expression increased in *wdr-23* mutants, transcripts of 810 were significantly reduced in the WDR-23 rescue animals (Figure 3A and Table S1). The average expression levels of the rescued genes in *wdr-23* mutants was 20.74 fold above wild type which is similar to the average of 23.99 for all 2,285 genes, indicating that the rescue was not caused by a bias generated by rescuing specifically low expressing genes in *wdr-23* mutants.

We used the Database for Annotation, Visualization and Integrated Discovery (DAVID) [28], to assign GO terms for the 810 rescued genes; of the 492 genes with predicted functions, many genes involved in stress, detoxification and metabolism were identified, consistent with the known stress response roles of *skn-1* (Figure 3B). The expression of 177 genes has been experimentally examined (wormbase.org), and 93 of these are expressed in the nervous system (Figure 3C and Table S1). A subset of these is expressed exclusively in neurons (Table 1), including eight genes encoding insulins or FMRFamide related peptides, as well as three genes encoding proteins involved in neuropeptide processing—*egl-3/*proprotein convertase, *egl-21/*carboxypeptidase, and *sbt-2/*7B2. We also identified five genes encoding synaptic proteins, including *unc-13/*Munc13, *nlg-1/*neuroligin, *pde-4/*cyclic nucleotide phosphodiesterase, *dlk-1/*MAPKKK, and *cab-1/*NPDC-1 [29]–[32]. Together, these results suggest that neuronal WDR-23, possibly through SKN-1, regulates the expression of genes involved in neuroendocrine signaling and synaptic function.

**Table 1:** Neuronal targets of SKN-1 identified by RNA sequencing.

Sequence	Gene	*wdr-23* RPKM	Rescue RPKM	*q* value	*Function*	WWTDTCAT Consensus
**Neurotransmitter receptor/channels**						
F53E10.2	*acr-17*	2.72	0.88	0.044	AcetylCholine Receptor	2
F59B1.9	*acr-23*	2.34	0.39	0.007	AcetylCholine Receptor	2
Y39A3B.5	*ckr-2*	4.93	2.14	0.038	CholecystoKinin Receptor homolog	0
C14F11.3	*lite-1*	14.73	2.73	0.000	high-energy LIghT unrEsponsive	3
F07F6.6	*nmr-1*	1.81	0.33	0.000	NMDA class glutamate receptor	0
F22B7.7	*twk-7*	10.86	4.14	0.032	TWiK family of potassium channels	1
K03B8.9	*deg-3*	5.28	1.76	0.011	DEGeneration of certain neurons	3
**Neuropeptide related**						
F01D4.4	*egl-21*	167.13	78.59	0.000	EGg Laying defective	1
F23B2.5	*flp-1*	91.90	40.17	0.003	FMRF-Like Peptide	3
F33D4.3	*flp-13*	45.08	17.05	0.028	FMRF-Like Peptide	2
Y37D8A.15	*flp-14*	61.85	29.67	0.038	FMRF-Like Peptide	0
Y48D7A.2	*flp-18*	68.87	29.16	0.019	FMRF-Like Peptide	1
C03G5.7	*flp-5*	81.68	30.02	0.009	FMRF-Like Peptide	1
F31F6.4	*flp-8*	52.69	19.64	0.008	FMRF-Like Peptide	0
C36H8.3	*flp-9*	94.50	39.40	0.007	FMRF-Like Peptide	3
ZK84.7	*ins-20*	115.05	3.18	0.000	INSulin related	3
C51E3.7	*egl-3*	181.88	71.98	0.000	Proprotein convertase	3
B0244.2	*ida-1*	79.04	36.08	0.002	related to Islet cell autoantigen	0
T03D8.3	*sbt-1*	178.81	86.52	0.009	7B2 prohormone convertase chaperone	2
**Presynaptic structure/function**						
C23H4.1	*cab-1*	159.33	82.03	0.000	C-terminus-of-AEX-Binding protein	2
F33E2.2	*dlk-1*	37.71	24.67	0.049	DAP Like Kinase	0
C40C9.5	*nlg-1*	14.06	5.68	0.001	NeuroLiGin family	5
R153.1	*pde-4*	48.15	30.29	0.036	PhosphoDiEsterase	1
ZK524.2	*unc-13*	19.60	10.98	0.002	UNCoordinated	2
**Signal transduction**						
B0412.2	*daf-7*	12.43	4.12	0.019	abnormal DAuer Formation	2
T13C5.1	*daf-9*	3.28	0.83	0.005	abnormal DAuer Formation	2
Y73F8A.1	*pkd-2*	3.76	0.68	0.000	human PKD2 related	2
C02C6.2	*olrn-1*	14.24	6.06	0.015	Olfactory LeaRNing defective	2
T13H10.1	*kin-5*	3.88	0.89	0.017	protein KINase	0
ZK265.5	*sre-23*	3.80	0.86	0.029	Serpentine Receptor, class E	5
**Other**						
F26D12.1	*fkh-7*	59.08	29.71	0.004	ForKHead transcription factor family	0
R06F6.6	*ceh-62*	18.94	7.35	0.038	Uncharacterized homeobox protein	1
D1046.5	*tpra-1*	46.32	19.46	0.000	UPF0359 membrane protein	2
F26A3.5		9.39	1.94	0.007		1
F59C6.2		4.49	0.49	0.001		2
Y32H12A.1		10.57	0.86	0.000		0
ZC21.3		35.45	15.18	0.013		0

RNAseq list of genes with neuronal expression patterns according to WormMart WS220 that were rescued by *Psnb-1::wdr-23a* in *wdr-23* mutants. Functions identified by DAVID. Consensus indicates the number of SKN-1 binding sites (WWTDTCAT) in promoter fragments within 1 kb of transcriptional start site as evaluated by RSAT.

To identify genes that may be direct SKN-1 targets, we examined the promoters of the neuronal genes for the presence of consensus SKN-1 binding sites. SKN-1 is predicted to bind the consensus sequence WWTDTCAT on either strand in the promoters of target genes. Using the web based program Regulatory Sequence Analysis Tools (RSAT), we scanned 1000 bp promoter fragments for each gene for the SKN-1 consensus [33]; the promoter fragments for 70 of the 93 neuronal genes contained at least one potential binding site (Table S1). We then cross-referenced the list of rescued neuronal genes with SKN-1 ChIP-seq datasets taken from L1, L3 and L4 stage animals (ModEncode and [34]) and found that 31 genes contained significant SKN-1 peaks within 2000 bp upstream or 500 bp downstream of the transcription start site (Table S1). These results indicate that a subset of the neuronal genes identified by RNAseq may be direct binding targets of SKN-1.

### *nlg-1* expression is positively regulated by SKN-1

Among the neuronal genes containing SKN-1 consensus sites in their promoter, the cell adhesion molecule *nlg-1/*neuroligin emerged as an interesting candidate since it has been implicated in *C. elegans* stress response and in synaptic function [30], [35], [36]. *nlg-1* is the sole neuroligin family ortholog in *C. elegans*, and NLG-1 is expressed in cholinergic motor neurons where it localizes to presynaptic terminals [35], [37].

**Figure 4:**
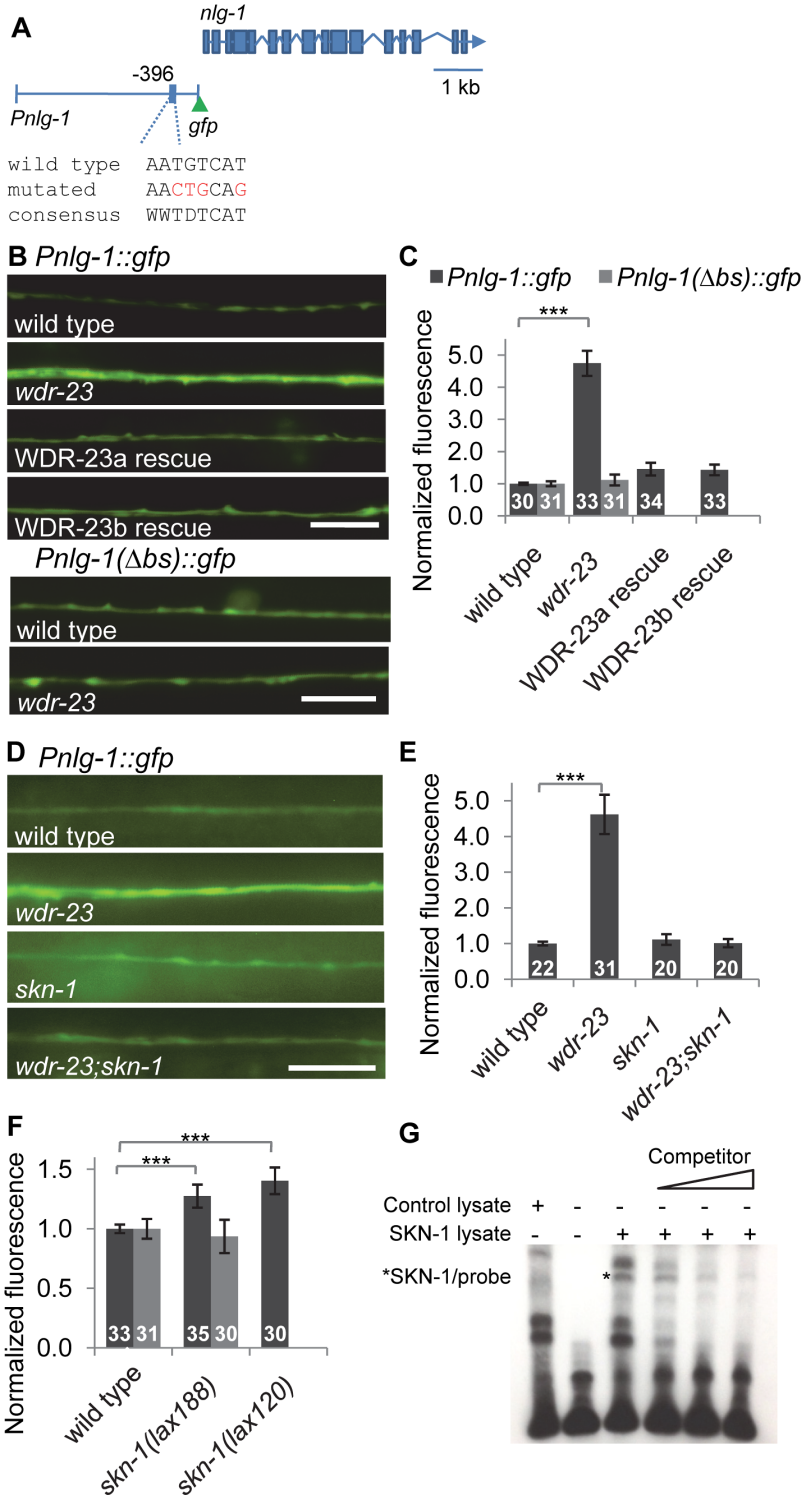
*nlg-1* transcription is regulated by neuronal SKN-1. A) Schematic of *Pnlg-1::gfp* reporters; *vjIs47* and *vjIs48* contain a 3.6 kb *nlg-1* promoter driving *gfp*. Location of SKN-1 binding site and mutated site indicated. B) *Top*, Representative images of *Pnlg-1::gfp* (*vjIs47*) expression in the ventral cord of indicated strains. WDR-23a and WDR-23b rescues indicate isoform specific cDNA driven by the *nlg-1* promoter (*vjEx447* and *vjEx436*, respectively) and expressed in *wdr-23* mutants. *Bottom*, Representative images of the extra-chromosomal transgene *Pnlg-1(*Δ*bs)::gfp* (*vjEx391*) in the indicated strains. C) Quantification of *Pnlg-1::gfp (vjIs47)* and *Pnlg-1(*Δ*bs)::gfp (vjEx391)* in indicated strains. Sample sizes indicated. Values normalized to wild type (*vjIs47*) or deleted binding site (*vjEx391*), respectively. D) Representative images of *Pnlg-1::gfp (vjIs48)* heterozygotes in indicated strains. *skn-1* indicates *zu67* loss of function allele, unless otherwise indicated. E–F) Normalized quantification of *Pnlg-1::gfp* (*vjIs48*) heterozygotes (E), *Pnlg-1::gfp (vjIs48)* homozygotes and *Pnlg-1(*Δ*bs)::gfp (vjEx756*, F) in indicated strains. All *nlg-1* reporters were imaged using the same microscope settings, and expression of reporters was similar between strains (total fluorescence *vjIs48* in wild type animals: 3280.6±155.6; total fluorescence of *vjEx756* in wild type animals: 2919.7±241.7). G) Electrophoretic mobility shift assay for binding of full-length SKN-1a to the consensus sequence at −396 bp. Lysate was either unprogrammed control or programmed to express SKN-1a. Competitor is 20, 100 and 200 fold molar excess of unlabeled probe. Scale bar represents 10 µm; error bars represent ±sem; ***p<0.001.

To examine the effects of *skn-1* on *nlg-1* expression, we constructed reporter strains consisting of a 3.6 kb *nlg-1* rescuing promoter fragment [30] driving soluble *gfp* (*Pnlg-1::gfp*, *vjIs47*, and *vjIs48*; Figure 4A). In wild type animals, we detected *Pnlg-1::gfp* fluorescence in a few head neurons and in ventral nerve cord neurons, previously reported to be DA and VA class cholinergic motor neurons [30], [35]. *nlg-1* reporter fluorescence has been reported at low levels in muscles [30], but we did not detect *Pnlg-1::gfp* in muscle cells in either transgenic line. In mutants lacking *wdr-23, Pnlg-1::gfp* fluorescence in motor neurons increased approximately 5 fold (Figure 4B and 4C), in agreement with the 5.6 fold increase in *nlg-1* transcript levels detected by RNAseq [20]. Cell-specific expression of either *wdr-23a* or *wdr-23b* cDNA in *nlg-1* expressing cells (using the *nlg-1* promoter) fully rescued the increased *Pnlg-1::gfp* fluorescence to wild type levels (Figures 4B and 4C), indicating that the regulation of *nlg-1* expression by *wdr-23* is cell autonomous.

In *skn-1* mutants lacking the *skn-1a/c* isoforms (*zu67* mutants), *Pnlg-1::gfp* expression was similar to wild type. However, *skn-1* is required for the increased *Pnlg-1::gfp* fluorescence caused by loss of *wdr-23*, since *skn-1* mutations reduced the *Pnlg-1::gfp* reporter fluorescence of *wdr-23* mutants to wild type levels (Figures 4D and 4E). Conversely, *Pnlg-1::gfp* fluorescence increased by ∼35% in mutants in which *skn-1* is hyperactive (*lax120gf* or *lax188gf*; Figure 4F). *lax120gf* and *lax188gf* are thought to prevent SKN-1a/c interaction with mitochondrial docking proteins, resulting in an activated pool of SKN-1a/c [38]. Together, these results show that *skn-1* is not necessary for baseline *nlg-1* expression in motor neurons, but *skn-1* activation positively regulates *nlg-1* expression.

The *nlg-1* promoter has four SKN-1 binding consensus sites within 500 bp upstream of the transcriptional start site (Figure S1). Promoter alignments between the nematode *Caenorhabditis* species *elegans*, *briggsae*, *japonica* and *remanei* revealed that one of these sites located 396 bp upstream of the *C. elegans* start is completely conserved in all species (Figure S1). This site has the sequence AATGTCAT, which matches the consensus perfectly. The underlined region is predicted to be a largely invariant sequence that directly interacts with SKN-1 [13], [39]. We mutated AATGTCAT at −396 to AACTGCAG in the *Pnlg-1::gfp* reporter to create a reporter with a deleted binding site (the *Pnlg-1(*Δ*bs)::gfp* reporter; Figure 4A). Basal motor neuron fluorescence of transgenic animals expressing *Pnlg-1(*Δ*bs)::gfp* was similar to transgenic animals expressing the *Pnlg-1::gfp* reporter (Figure 4B). However, *Pnlg-1(*Δ*bs)::gfp* reporter fluorescence did not increase in either *wdr-23* mutants or in *skn-1(gf)* mutants compared to wild type controls (Figure 4B, 4C and 4F), suggesting that this site is critical for *skn-1-*mediated increases in *nlg-1* expression. To test whether SKN-1 binds to this site, we performed electrophoretic mobility shift assays. *In vitro* translated SKN-1a bound to labeled probes containing the putative SKN-1 binding site in the *nlg-1* promoter, and binding was disrupted by the addition of excess unlabeled probe (Figure 4G). These results indicate that the SKN-1 binding site at −396 in the *nlg-1* promoter can be bound by SKN-1 *in vitro* and is critical for *skn-1-*induced expression of *nlg-1 in vivo*.

### Synaptic abundance of NLG-1/neuroligin levels is regulated by SKN-1 signaling

**Figure 5:**
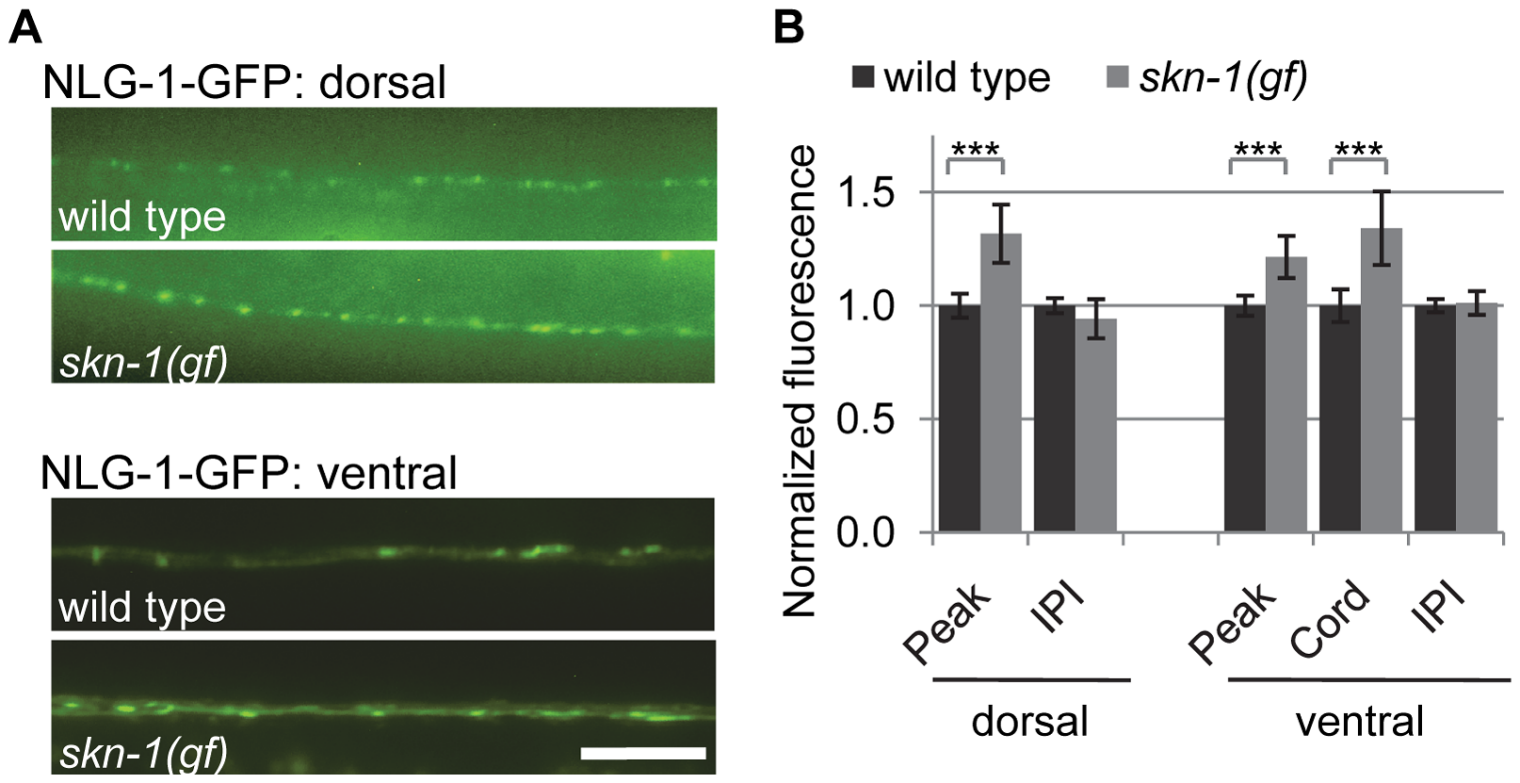
Synaptic NLG-1 levels are regulated by SKN-1. A) Representative images of *Pnlg-1::nlg-1-gfp* (*vjIs105*) in the dorsal (*top*) and ventral (*bottom*) cords of the indicated strains. *skn-1(gf)* indicates *lax188* gain-of-function allele. B) Quantification of punctal fluorescence (peak), cord fluorescence (cord) and interpunctal interval (IPI) of *Pnlg-1::nlg-1-gfp (vjIs105)* in dorsal (wild type n = 31, *skn-1(gf)* n = 29) and ventral cords (wild type n = 32, *skn-1(gf)* n = 26) of the indicated strains. Cord fluorescence could not be measured for *vjIs105* in the dorsal cord because it was not above background levels. Scale bar represents 10 µm; error bars represent ±sem; ***p<0.001.

To determine whether the transcriptional regulation of *nlg-1* by SKN-1 impacts NLG-1 protein levels at synapses, we examined synaptic levels of NLG-1 in animals expressing a fusion protein in which GFP was inserted near the C-terminus of NLG-1 (NLG-1-GFP, *vjEx561* and *vjIs105*). This fusion protein is functional [30] and localizes to presynaptic terminals in motor neurons [35], [37]. NLG-1-GFP driven by the *nlg-1* promoter adopted a punctate pattern of fluorescence in the dorsal and ventral cords, where presynaptic terminals of DA and VA class motor neurons are located, respectively (Figure 5A). We examined changes in the synaptic abundance of NLG-1-GFP by measuring the average punctal fluorescence intensity (peak fluorescence) and synapse number (interpunctal interval) [40], [41]. In *skn-1(gf)* mutants, the punctal fluorescence of NLG-1-GFP significantly increased in both the dorsal and ventral cords, while the interpunctal interval did not change (Figure 5A, 5B and Table S2). These results indicate that SKN-1 positively regulates synaptic NLG-1 protein abundance but does not affect synapse number.

### Environmental stress induces NLG-1 expression

**Figure 6:**
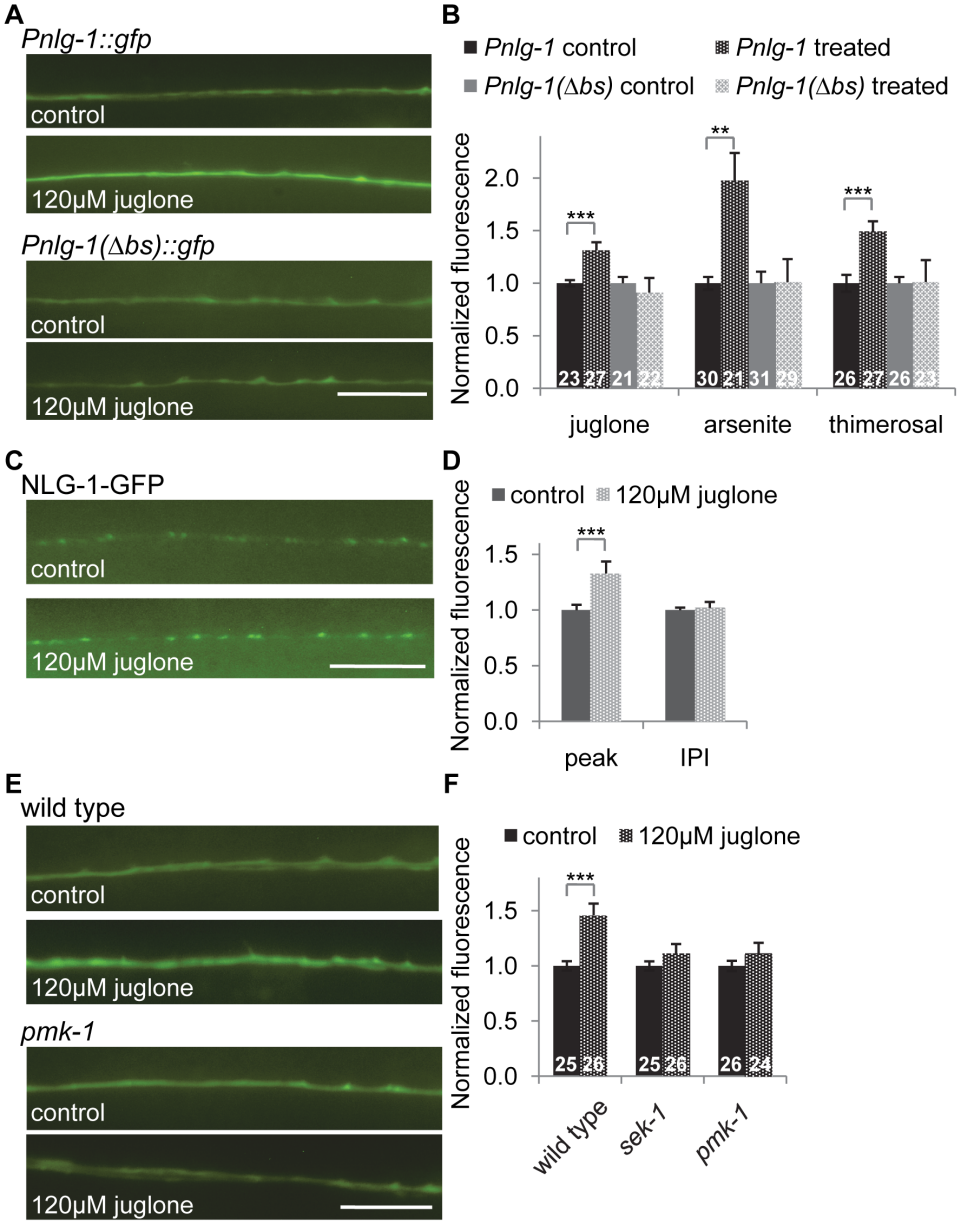
*nlg-1* transcription is regulated by the SKN-1 pathway. A–B) Representative images (A) and quantification (B) of animals expressing the *Pnlg1::gfp* (*vjIs47)* or *Pnlg-1(Δbs)::gfp* (*vjEx756*) reporters exposed to control, 120 µM juglone, 5.0 mM sodium arsenite or 35 µM thimerosal. L4 stage animals were exposed to drug overnight for 14 hours, followed by 2–4 hour recovery prior to imaging. Values of stressed animals were compared to non-stressed animals of the same genotype to determine significant changes in response to stress. Sample sizes indicated. C–D) Representative images (C) and quantification (D) of *Pnlg-1::nlg-1-gfp* (*vjIs105*) in the dorsal cord of animals exposed to control (n = 30) or 120 µM juglone (n = 29) treatments. E–F) Representative images (E) and quantification (F) of *Pnlg-1::gfp* (*vjIs47*) in indicated strains exposed to control or 120 uM juglone. Sample sizes indicated. Scale bar represents 10 µm; error bars represent ±sem; **p<0.01, ***p<0.001.

We next tested whether toxins that induce oxidative stress could increase neuroligin expression and abundance in motor neurons. We found that exposure to the mitochondrial stressors juglone or sodium arsenite, both of which have been shown to activate *skn-1* [42], [43], robustly induced *Pnlg-1::gfp* fluorescence in motor neurons compared to untreated animals (Figure 6A and 6B). Treatment with an organic mercury (thimerosal) increased nuclear SKN-1::GFP in the intestine (Figure S2) and also increased *Pnlg-1::gfp* fluorescence (Figure 6B). However, these toxins had no effect on *Pnlg-1(*Δ*bs)::gfp* fluorescence (Figure 6A and 6B). We found that juglone treatment significantly increased punctal fluorescence of NLG-1-GFP, without changing the interpunctal interval (Figure 6C, 6D and Table S2). These results suggest that activation of *skn-1* by oxidative stress increases NLG-1 synaptic abundance by increasing *nlg-1* expression in neurons.

### Genetic regulation of *nlg-1* expression

To further explore how SKN-1 activity is regulated in the nervous system, we tested the impact of altering insulin signaling, mitochondrial respiration or synaptic activity on *Pnlg-1::gfp* expression. The insulin-like signaling (IIS) pathway regulates SKN-1 in the intestine [44]; activation of DAF-2/insulin-like receptor leads to SKN-1 phosphorylation by SGK-1/SGK, resulting in decreased SKN-1 activity. We examined putative null *sgk-1* mutants and found that *Pnlg-1::gfp* reporter fluorescence increased by ∼25% (Figure S3). Conversely, during conditions of stress, PMK-1/p38 MAPK phosphorylates SKN-1, resulting in nuclear SKN-1 translocation [45]. We found no change of baseline *Pnlg-1::gfp* fluorescence in mutants lacking either *sek-1/*MAPKK or *pmk-1* (Figure 6E, 6F and S3). However, *sek-1* and *pmk-1* mutations suppressed the juglone-induced increase in *Pnlg-1::gfp* reporter fluorescence (Figure 6E and 6F).

*C. elegans* mutants with impaired mitochondrial respiration, for example the conserved *clk-1/*COQ7, necessary for the biosynthesis of coenzyme Q, and *isp-1/*ISP, a subunit of mitochorial complex III, are resistant to toxins that increase oxidative damage [46]. *pink-1/*PINK1, on the other hand, is predicted to act in conjunction with the E3 ligase Parkin to initiate mitophagy of damaged mitochondria [47], [48]. *Pnlg-1::gfp* expression increased mildly in mutants lacking *clk-1*, but not *isp-1*. Conversely, loss of *pink-1* resulted in a significant decrease of *Pnlg-1::gfp* fluorescence (Figure S3).

Finally, in order to test whether neuronal activity regulates SKN-1 in the nervous system, we examined mutants with increased synaptic activity (*dgk-1/*diacylglycerol kinase or *goa-1/*Gαo) and mutants with decreased activity (*unc-2/*VGCC or *unc-18/*nSec1), as well as mutants lacking *mef-2/*MEF and *mir-1/*microRNA, which are involved in a retrograde synaptic signaling pathways that is dependent on *nlg-1* [35]. We detected no change in reporter fluorescence in these mutants (Figure S3). Together, these results indicate that insulin signaling and mitochondrial metabolism contribute to the activation of SKN-1 in neurons, while changes in synaptic transmission do not seem to impact neuronal SKN-1 activity.

### NLG-1 promotes organismal survival during SKN-1-dependent response to mitochondrial stress

**Figure 7:**
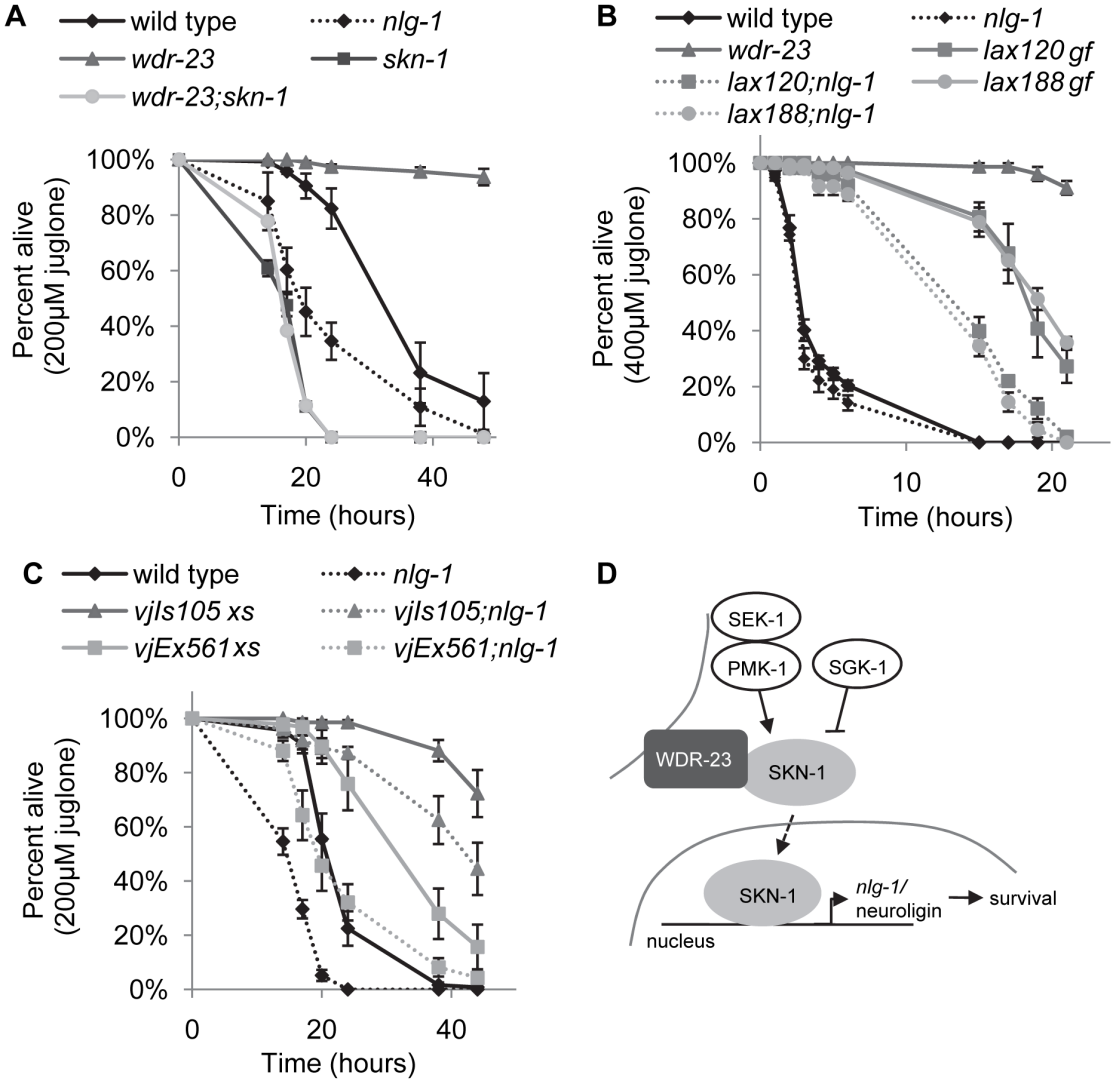
NLG-1 is protective to juglone. A–B) Survival curves of indicated strains on 200 uM juglone. C) Survival curve of indicated strains on 400 uM juglone. D) Model for SKN-1-dependent transcription of *nlg-1*. Stress activated the SKN-1 pathway, which increases *nlg-1* expression; increased NLG-1 activity promotes organism survival. Error bars represent ±sem. Log rank tests were used to identify significant differences between genotypes and reported in Table S3. Curves depict average values for four replicates of n = 40 per genotype.

We next sought to determine whether *nlg-1* mediates the protective effects of *skn-1* activation in response to environmental toxins. *skn-1* mutants are hypersensitive to toxicity induced by arsenite and juglone treatment (Figure 7A and S4; [42], [43]). In addition, we found that *skn-1* mutants were sensitive to thimerosal-induced toxicity (Figure S5), in agreement with studies showing SKN-1 protects from metal toxicity [15], [18]. In contrast, animals lacking *wdr-23* were resistant to toxicity of both thimerosal and juglone, and resistance was completely blocked by loss of *skn-1* (Figures 7A and S5; [43]). Similarly, hyperactive *skn-1(gf)* mutants were resistant to juglone toxicity (Figure 7B and S6). Interestingly, loss of *wdr-23* did not confer protection against sodium arsenite (Figure S4), suggesting specificity in drug resistance in mutants lacking *wdr-23*.

*nlg-1* mutants are hypersensitive to heavy metal toxicity by thimerosal and oxidative stress induced by paraquat [30]. We found that mutants lacking *nlg-1* were also more sensitive to juglone toxicity (Figure 7A and S5). In contrast, transgenic animals over-expressing NLG-1-GFP were significantly more resistant to juglone-induced toxicity compared to non-transgenic controls (Figure 7C). To confirm that the juglone resistance caused by NLG-1-GFP transgenes was due to *nlg-1* expression, we examined juglone responses of animals expressing *Pnlg-1::gfp* and found that they were not as resistant to juglone as NLG-1-GFP expressing animals (Figure S7). Finally, *nlg-1* mutations dramatically reduced the ability of activated *skn-1* to protect animals from the toxic effects of juglone: *skn-1(gf);nlg-1* double mutants were significantly less resistant to juglone than *skn-1(gf)* mutants alone (Figure 7B). Together, these data suggest that the dosage of *nlg-1* is a critical determinant of survival in response to stress, and that *nlg-1* contributes to *skn-1-*dependent survival.

## Discussion

In this study, we find that WDR-23 in neurons, possibly acting through SKN-1, regulates the expression of approximately 800 genes in multiple tissues, including the nervous system. One of these genes, the synaptic cell adhesion molecule *nlg-1/*neuroligin, contains a consensus SKN-1 binding site in its promoter necessary for SKN-1 binding *in vitro* and for *nlg-1* expression following SKN-1 activation. Increasing SKN-1 activity elevates the abundance of NLG-1 protein at synaptic terminals, and changes in synaptic NLG-1 levels correlate with altered survival of animals in the presence of mitochondrial toxins. We propose a model in which, in response to mitochondrial stress, SKN-1 induces transcription of *nlg-1*, which in turn enhances organismal survival (Figure 7D).

### SKN-1 transcriptional targets in neurons

Previous studies have demonstrated SKN-1/Nrf2 dependent transcriptional programs are initiated in response to oxidative and xenobiotic stress, and these programs are critical for organismal survival and longevity [42], [43], [49]. Among the genes regulated by SKN-1, few have been shown to mediate the protective effects of SKN-1, and fewer still have been shown to be direct binding targets. Furthermore, attempts to identify a comprehensive set of genes regulated by SKN-1/Nrf2 required to protect organisms from stress have been difficult due to the lack of tissue-level resolution.

Here, we have used comparative whole transcriptome RNA sequencing to identify an inclusive set of genes that are likely to be regulated by SKN-1 in the nervous system. For this analysis, we examined *wdr-23* mutants expressing a rescuing *wdr-23a* transgene driven by the pan-neuronal *snb-1* promoter. Genetic studies indicate that several distinct phenotypes displayed by *wdr-23* mutants are completely suppressed by *skn-1*, indicating that SKN-1 is selectively activated in *wdr-23* mutants, and a yeast-two hybrid screen identifies the only binding target of WDR-23 to be SKN-1 [19], indicating that SKN-1 is selectively activated in *wdr-23* mutants. Interestingly, *wdr-23* mutants appear to activate SKN-1 to a greater extent than *skn-1(gf)* mutations or toxin treatment. For example, loss of *wdr-23* results in greater survival in response to juglone treatment than *skn-1(gf)* mutations. In addition, *wdr-23* mutations increase *nlg-1* expression in the ventral cord neurons fivefold, whereas *skn-1(gf)* or toxin treatment increased it by approximately 30%. Thus, *wdr-23* mutants may provide increased sensitivity when used for transcriptional profiling, maximizing our ability to identify SKN-1 targets expressed in low abundance or in a small subset of cells. It is, however, possible that WDR-23 has functions beyond SKN-1 regulation, in which case some of the genes identified by this approach may not be targets of SKN-1. While the *snb-1* promoter fragment drives expression of GFP strongly in the nervous system, the possibility that this promoter may be leaky raises the prospect that some of the genes identified here may be regulated by WDR-23 in other tissues in addition to the nervous system.

The 810 genes identified here most likely represent either direct SKN-1 targets or indirect targets of SKN-1 that are secondarily activated in neurons or other tissues. SKN-1 ChIP-seq of larval stage animals identified a list of approximately 3000 genomic peaks bound by SKN-1 *in vivo* [34]; we cross-referenced our gene list of rescued neuronal genes with SKN-1 ChIP-seq datasets taken from L1, L3 and L4 stage animals [34] and found that a subset of the genes we identified contain a significant SKN-1 peak near to their transcriptional start sites (Table S1), but many genes do not. Differences between these datasets may be a byproduct of using non-stressed animals for the ChIP-seq experiments, reflecting basal, but not stress-induced, promoter occupancy by SKN-1.

Among the genes we identified were several neuropeptides and insulins, as well as peptide processing enzymes reported to be expressed in neurons. We speculate that stress-induced peptide processing and release may be part of a humoral response to promote organismal survival. Identification of the precise peptidergic signaling pathways will help to elucidate the mechanisms by which SKN-1 confers survival. We also identified a handful of known synaptic genes, including two cell adhesion molecules, *nlg-1* and *ncam-1*, indicating SKN-1 might play a role in maintaining the stability of neural networks. A theory has emerged suggesting axonal retraction might precede neuronal apoptosis in neurodegeneration, called dying back degeneration [50], and synaptic breakdown may occur prior to axonal retraction. The identification of cell adhesion molecules in this study suggests that prior to synaptic breakdown, neurons might initiate transcriptional programs to protect the integrity of the synapse. In support of this, recent evidence suggests that increased expression of NCAM in SH-SY5Y cultures prevents oxidative stress-induced apoptosis, and over-expression of a truncated NCAM molecule protects neuronal tissue in lesioned rats [51], [52].

### SKN-1 as a mitochondrial stress sensor

Activation of SKN-1/Nrf2 can protect against cell toxicity induced by oxidative stress [53]–[55]. In primary neuronal cultures, for example, activation of Nrf2 prevents cell death in response to rotenone and MPP, potent inhibitors of mitochondrial respiration [11]. Loss of OPA1, a key regulator of the morphology of mitochondria, results in increased Nrf2 activation [56]. These studies and others collectively suggest a potential role for SKN-1/Nrf2 as a sensor for increased mitochondrial dysfunction in the nervous system. We found that genes involved in drug detoxification, including the glutathione precursors *gst-4*, *gst-10*, *gcs-1*, and *gst-1* were reduced by neuronal expression of *wdr-23a*. Interestingly, *gst-1* has also been shown to contribute to dopaminergic neuron survival after manganese treatment [16]. Given their confirmed expression in neurons, is possible that these genes have a role in directly enhancing neuronal protection.

SKN-1 associates with purified mitochondrial fractions, and the *skn-1(gf)* alleles are proposed to reduce mitochondrial association, suggesting that mitochondria may act as a sink for SKN-1 [38]. Our data is consistent with the idea that WDR-23a associates with presynaptic organelles, including mitochondria. First, WDR-23a localizes to presynaptic terminals, where mitochondria are abundant. Second, WDR-23a remains associated with mitochondria in mutants in which mitochondria are displaced. Third, WDR-23a localizes to the outer membrane of mitochondria in muscle cells [20]. We speculate that WDR-23 may be a mitochondrial stress sensor that regulates SKN-1 abundance.

### Neuroligin as a neuronal target of SKN-1

Our data supports the idea that SKN-1 activation leads to increased NLG-1 abundance at synapses. We found that *nlg-1* confers some, but not all, of the protective effects of activation of SKN-1, as *nlg-1* mutations reduced, but did not eliminate, the resistance of *skn-1(gf)* mutants to juglone. Additional SKN-1 targets either in neurons or in other tissues are likely to contribute to organismal survival in response to juglone treatment. Interestingly, loss of *nlg-1* did not suppress *wdr-23* mutants resistance to thimerosal or juglone (Figure S5 and data not shown). This may be due to higher SKN-1 activity in *wdr-23* mutants compared to *skn-1(gf)* mutants, which may compensate for the lack of *nlg-1*. Because *nlg-1* mutants themselves are hypersensitive to stress, it is possible that *nlg-1* and *skn-1* function in parallel pathways to promote resistance; however, our data do not support this idea, but rather support the notion that *nlg-1* is a direct transcriptional target of SKN-1. Basal *nlg-1* transcription is not likely to be under *skn-1* regulation, since *nlg-1* reporter expression remains unchanged in *skn-1* mutants. Consistent with this, SKN-1 does not occupy the SKN-1 binding site we identified at position −396 in the *nlg-1* promoter in unstressed larval animals by ChIP-seq analysis.

How might increased expression of a synaptic cell adhesion molecule promote organismal survival in response to stress? Recent work has established the presence of “mitokines” in neurons—a signal produced in the neurons in response to dysfunctional mitochondrial electron transport [57]; release of these mitokines results in increased organismal survival. It is possible that *nlg-1* may be required in neurons for proper release of mitokines. Furthermore, the neuropeptides identified in this study are candidates for being signals released in response to stress to promote resistance. *nlg-1* expression has been detected in head neurons, motor neurons, and muscle cells [30]; thus, it is possible that *nlg-1* functions in any of these tissues to convey protection.

In mammals, neuroligin is a post-synaptic cell adhesion molecule that binds to the presynaptic protein neurexin; this junction is necessary for maintaining mature synaptic connections and normal synaptic transmission [58], [59]. In humans, rare mutations in neuroligin are associated with autism and other cognitive disorders. Some of these mutations reduce neuroligin delivery to the cell surface, interfering with synapse development and synaptic transmission [60]–[62]. Interestingly, it has been suggested that oxidative stress and mitochondrial dysfunction may play a role in the pathogenesis of autism, as certain biomarkers for oxidative stress are elevated in autistic patients [63]–[65]. It is possible that individuals with these mutations are unable to increase synaptic neuroligin levels in response to stress, and it will be interesting to identify the cellular and molecular mechanisms underlying neuroligin-dependent survival in response to stress.

## Materials and Methods

### *C. elegans* strains

Strains were cultured at 20° using standard methods. All experiments were performed on young adult hermaphrodites unless otherwise indicated. The following strains were provided by the Caenorhabditis Genetics Center, which is funded by the NIH National Center for Research Resources (NCRR): *sek-1(km4)*, *pmk-1(km25)*, *sgk-1(ok538)*, *skn-1(zu67)*, *nlg-1(ok259)*, *clk-1(e2519)*, *mef-2(gv1)*, *mir-1(gk276)*, *isp-1(gm150)*, and *pink-1(ok3538)*. Strain *wdr-23(tm1817)* was provided by the National BioResource Project (Japan). The wild type reference strain was N2 Bristol. The following strains were also used: *drp-1(tm1108), dgk-1(nu62), goa-1(sa734), unc-2(lj1), unc-18(md299), glo-1(zu391), unc-104(e1265), skn-1(lax120gf), skn-1(lax188gf), nuIs152*[*Pttx-3::RFP, Punc-129::GFP::SNB-1*]II, *nuIs321*[*Pmyo-2::GFP, Punc-17::mCherry*], *nuIs225*[*Pmyo-2::GFP, Psnb-1::WDR-23a*], *idIs7*[*rol-6(su1006), Pskn-1::skn-1b/c::GFP*], *yuIs25*[*Pmyo-2::GFP, Punc-129::mito-GFP*]V, and *vjIs26*[*Pmyo-2::GFP, Punc-129::WDR-23a::GFP*]III. Mutant strains were outcrossed a minimum of 4 times; all integrants were outcrossed at least 8 times.

### Molecular biology

*C. elegans* cDNA was used to clone all genes into pPD49.26 using standard molecular biology techniques, unless otherwise noted. Promoter elements were amplified from mixed stage genomic DNA. The following plasmids were generated: pTS147[*Punc-129::invom::rfp*], pTS31[*Pbec-1::nls-gfp*], pDS284[*Pnlg-1::gfp*], pDS286[*Pnlg-1(*Δ*BS)::gfp*], pKG8[*Pnlg-1::nlg-1-gfp*], pTS79[*Punc-17::skn-1a::gfp*], pDS139[*Punc-129::snb-1::mCherry*], pDS237[*Psnb-1::wdr-23a::gfp*], pTS85[*Psnb-1::wdr-23b::gfp*], pDS334[*Pnlg-1::wdr-23a*], pDS335[*Pnlg-1::wdr-23b*], and pTS199[T7::skn-1a].

Oligo sequences:

*Pbec-1*

oTS44: ccccccGGATCCcgacaattatacatgttcccc

oTS45: ccccccGCTAGCcgactgactggattatgatagatcc

*Pnlg-1*

oDS694: ccccccGCATGCtaagcccccgtacgctaacacc

oXL13: ccccccGGATCCgcctgttcacttccaaattcgc

*Pnlg-1(*Δ*bs)*

oDS692: cctgttgccccccaaatgCTGCAGttacctcttttcctcccttctacc

oDS693 ggtagaagggaggaaaagaggtaaCTGCAGcatttggggggcaacagg

### Transgenic lines

Transgenic strains were generated by injecting N2 with expression constructs (2.5–90 ng/µL) and the co-injection marker KP#708 (*Pttx-3::rfp*, 40 ng/µL) or KP#1106 (*Pmyo-2::gfp*, 10 ng/µL). Microinjection was performed using standard techniques as previously described [66]. At least three lines for each transgene were examined for expression, and representative lines were quantified. The following strains were made: *vjEx7*[*Punc-129::wdr-23b::gfp*] *vjEx663*[*Punc-129::invom::rfp*], *vjEx254*[*Pbec-1::nls-gfp*], *vjEx391*[*Pnlg-1(*Δ*bs)::gfp*], *vjEx756*[*Pnlg-1(*Δ*bs)::gfp*], *vjEx561*[*Pnlg-1::nlg-1-gfp*], *vjEx339*[*Punc-129::snb-1::mCherry*], *vjEx423*[*Psnb-1::wdr-23a::gfp*], *vjEx426*[*Psnb-1::wdr-23b::gfp*], *vjEx447*[*Pnlg-1::wdr-23a*], *vjEx436*[*Pnlg-1::wdr-23b*], *vjIs45*[*Punc-17::skn-1a::gfp*]II, *vjIs47*[*Pnlg-1::gfp*]IV, *vjIs48*[*Pnlg-1::gfp*]I, *vjIs105*[*Pnlg-1::nlg-1-gfp*]III.

### Microscopy and analysis

To image animals, adult worms were paralyzed using 2,3-butanedione monoxime (BDM, 30 µg/µL; Sigma) and mounted on 2% agarose pads for imaging. Images were captured with a Nikon eclipse 90i microscope equipped with a Nikon PlanApo 60× or 100× objective (NA = 1.4) and a PhotometricsCoolsnap ES2 camera. For fluorescence imaging of synapses, images were captured either from the ventral or dorsal cord near the posterior gonadal bend of the worm, as indicated. Images of animals expressing *nlg-1* reporters were captured at the ventral cord near the posterior gonadal bend of the worm. We found that *skn-1* mutants expressing fluorescent integrants arrest at larval stages when the integrant is homozygous, making quantification of fluorescence markers challenging; however, *skn-1* mutants expressing heterozygous integrants develop fully into adults.

Metamorph 7.0 software (Universal Imaging/Molecular Devices) was used to capture serial image stacks, and the maximum intensity projection was used for analysis of the dorsal and ventral cords. Line scans of the maximum intensity projection image were also recorded using Metamorph. The fluorescence intensity values were then quantified using Puncta 6.0 software written with Igor Pro (Wavemetrics), as previously described [67]. For all experiments, fluorescence values were normalized to the values of 0.5 µm FluoSphere beads (Invitrogen) captured during each imaging session. This was performed to provide a standard for comparing absolute fluorescence levels between animals from different sessions.

To quantify changes in neuronal SKN-1a::GFP, an anterior and posterior image was taken for each animal in both RFP and GFP channels. Cell bodies were identified by the presence of soluble mCherry. Average cell body fluorescence was calculated by outlining the entire cell body and taking the average intensity. Background values were determined by finding the average fluorescence of the area immediately adjacent to the cell body and were subtracted from each cells' average fluorescence. Cells were categorized as low, medium, or high expressing cells using arbitrary cut off levels after background subtraction—low expressing cells were those cells below the level of detection (less than 30 units different than background fluorescence), medium expressing cells were between 30–70 arbitrary units, and high expressing cells had a total fluorescence greater than 70 units.

### Toxicity assays

Stock solutions of 50 mM juglone (Calbiochem) and 20 mM thimerosal (Enzo) were freshly dissolved in DMSO or water, respectively, prior to addition to molten NGM. Sodium arsenite (Ricca) was maintained in aqueous solution at 0.5% w/v and stored at room temperature. Plates were freshly made approximately 24 hours before use and seeded with concentrated OP50 the night before being used.

To assess longevity, age matched young adult animals were transferred to 100 mm NGM plates containing either drug or control and assayed over time. Animals which escaped the plates were excluded from the analysis. At least four replicates of n = 40 animals per genotype per stress were tested; final samples sizes reported in Table S3. Animals were stored at 20° except during time points. Animals were scored as dead if they did not respond to repeated light prodding. Percentages alive for each genotype were determined by averaging the fraction alive per plate at each time point and plotting graphically.

For fluorescence toxicity studies using the *nlg-1* reporters, L4 stage animals were exposed to NGM plates supplemented with drug or control overnight for 14 hours; animals were allowed 2–4 hours recovery time before imaging. Concentrations were chosen that did not result in animal death after 14 hours. For juglone imaging, control plates were supplemented with an equal volume of DMSO, as the juglone was diluted to 50 mM in DMSO prior to addition to the test plates.

### Statistical analysis

A Student's *t* test was used to determine significance when comparing fluorescence of *nlg-1* reporters in different conditions, unless otherwise specified. Log rank tests with a Bonferroni correction were calculated by JMP Pro version 10.0 software and were used to determine significant differences between genotypes for toxicity studies; differences between genotypes are reported in Table S3.

### RNA sequencing

Total RNA was isolated from approximately 10,000 mixed stage animals for wild type, *wdr-23(tm1817)* mutants and *wdr-23;nuIs225* using Stat60 (Tel-test B, Texas). Transcriptome libraries were prepared using TruSeq RNA sample preparation kit (Illumina) according to manufacturer's TruSeq protocol as previously described [20]. Libraries were amplified by PCR and quality and quantity of libraries were evaluated on BioAnalyzer 2100 (Agilent). Sequencing was performed on HiSeq2000 (Illumina). Sequencing reads were aligned to the *C. elegans*genome (release WS210) using TopHat [68]. Gene models were downloaded from ENSEMBL and quantified using Cufflinks. Differentially expressed genes at false discovery rate (FDR) of 0.05 were identified using the Cuffdiff module of the Cufflinks package.

### Electrophoretic mobility shift assays

Full-length SKN-1a cDNA was cloned into a pBS backbone driven by the T7 promoter and expressed using TnT T7 Quick Coupled Transcription/Translation System for DNA (Promega). EMSA was performed using the LightShift Chemiluminescence EMSA Kit (Pierce) according to manufacturer's protocols. Complementary 5′ biotinylated oligonucleotides containing the *pnlg-1* SKN-1 binding site were self-annealed and incubated with 1 µl of SKN-1 lysate, 1 µg Poly (dIdC) and 5 mM MgCl_2_ for 20 minutes at room temperature. Samples were separated on a 5% native polyacrylamide gel and blotted on Biodyne B nylon membranes (Pierce).

Pnlg-1 probes:

oTS300: 5′ biotin-gttgccccccaaatgATGACATTacctcttttcctccc 3′

oTS310: 5′ biotin-gggaggaaaagaggtAATGTCATcatttggIgggcaac 3′

## Supporting Information

Figure S1 *nlg-1* promoter alignment for *Caenorhabditis* species. ***Indicates transcriptional start aligned for all four species. Consensus sequences (WWTDTCAT) were identified by RSAT and indicated for *C. elegans* by bold, underlined sequences. Conserved SKN-1 consensus sequence boxed.
(TIF)

Figure S2 Thimerosal increases intestinal SKN-1::GFP. Intestinal fluorescence of SKN-1b/c::GFP in control animals (top) compared to animals treated with 35 uM thimerosal for 14 hours. Animals were imaged at L3/L4 stage to limit background fluorescence.
(TIF)

Figure S3 Quantification of *Pnlg-1::gfp* (*vjIs47*) in the ventral cord of animals in different genetic backgrounds. Samples sizes shown. *p<0.05, **p<0.01.
(TIF)

Figure S4 Survival in response to sodium arsenite. Survival curves of indicated strains on 2.5 mM sodium arsenite. Error bars represent ±sem.
(TIF)

Figure S5 Survival in response to thimerosal. Survival curves of indicated strains on 50 µM thimerosal. Error bars represent ±sem.
(TIF)

Figure S6 Survival of *skn-1(gf)* on 200 µM juglone. Survival curves of indicated strains on 200 µM juglone. Error bars represent ±sem.
(TIF)

Figure S7 Survival of *Pnlg-1::gfp* on 200 µM juglone. Survival curves of indicated strains on 200 µM juglone. Error bars represent ±sem.
(TIF)

Table S1 RNA sequencing of *wdr-23* mutants and WDR-23 neuronal rescue. RNAseq list summarizing the 2,285 genes up-regulated in *wdr-23* mutants. Neuron rescue denotes *wdr-23* mutants expressing an integrated *wdr-23a* driven by the *snb-1* promoter (*vjIs225*). Genes highlighted in green are statically different between *wdr-23* mutants and WDR-23 rescue. Expression was identified for genes which are significantly different between *wdr-23* and WDR-23 rescue using WormMart. WWTDTCAT denotes SKN-1 binding sites identified within 1000 bp of gene start. Genes located proximally to ChIP-seq sites are identified by stage [34]; sites were restricted to 2 kb upstream or 500 bp downstream of start sites. Genes identified by microarrays are identified in Ref column as follows: *skn-1* RNAi^1^, exposure to arsenite^2^, exposure to *t*-butyl hydrogen peroxide^3^ [42] or hyperbaric oxygen^4^ [49].
(XLSX)

Table S2 Punctal analysis of NLG-1-GFP fluorescence. Raw values ± sem reported for peak, cord, width, and interpunctal interval (IPI).
(PDF)

Table S3 Statistical analysis of toxicity assays. Samples sizes, χ^2^ values for log rank tests, and significant differences between samples reported. Statistically significant differences were calculated using the log rank test with a Bonferroni correction to establish the thresholds of significance.
(PDF)
