# Correction: Regulation of Synaptic *nlg-1*/Neuroligin Abundance by the *skn-1*/Nrf Stress Response Pathway Protects against Oxidative Stress

**DOI:** 10.1371/journal.pgen.1004361

**Published:** 2014-04-10

**Authors:** The PLOS Genetics Staff

The second author’s name is spelled incorrectly. The correct name is: Oleg Evgrafov. The correct citation is: Staab TA, Evgrafov O, Knowles JA, Sieburth D (2014) Regulation of Synaptic *nlg-1*/Neuroligin Abundance by the *skn-1*/Nrf Stress Response Pathway Protects against Oxidative Stress. PLoS Genet 10(1): e1004100. doi:10.1371/journal.pgen.1004100
