# Supplementary figures and images for: Regulation of Synaptic nlg-1/Neuroligin Abundance by the skn-1/Nrf Stress Response Pathway Protects against Oxidative Stress
Source: PLoS Genet. 2014 Jan 16;10(1):e1004100. doi: 10.1371/journal.pgen.1004100 (PMC3894169; doi:10.1371/journal.pgen.1004100)

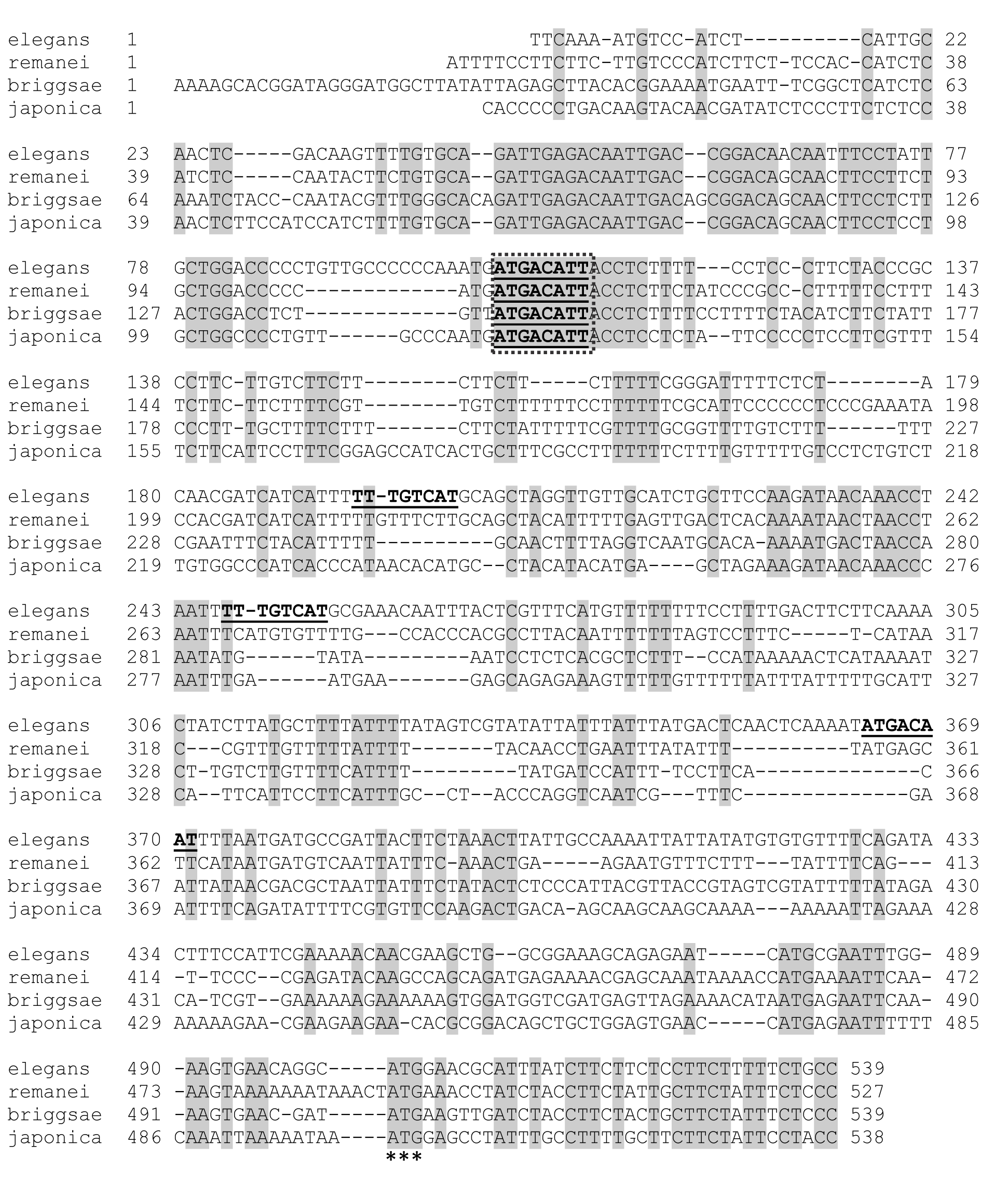

Supplement: Figure S1 — nlg-1 promoter alignment for Caenorhabditis species. ***Indicates transcriptional start aligned for all four species. Consensus sequences (WWTDTCAT) were identified by RSAT and indicated for C. elegans by bold, underlined sequences. Conserved SKN-1 consensus sequence boxed. (TIF) [file pgen.1004100.s001.tif]

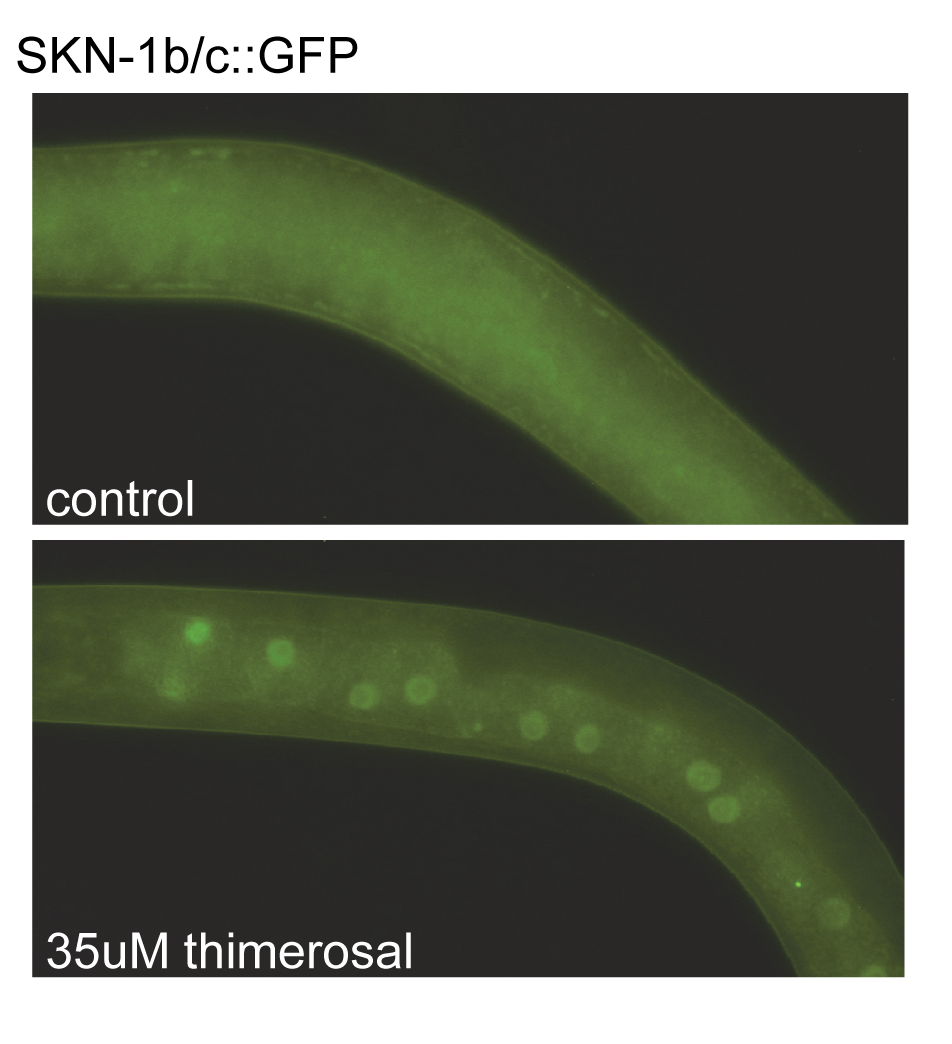

Supplement: Figure S2 — Thimerosal increases intestinal SKN-1::GFP. Intestinal fluorescence of SKN-1b/c::GFP in control animals (top) compared to animals treated with 35 uM thimerosal for 14 hours. Animals were imaged at L3/L4 stage to limit background fluorescence. (TIF) [file pgen.1004100.s002.tif]

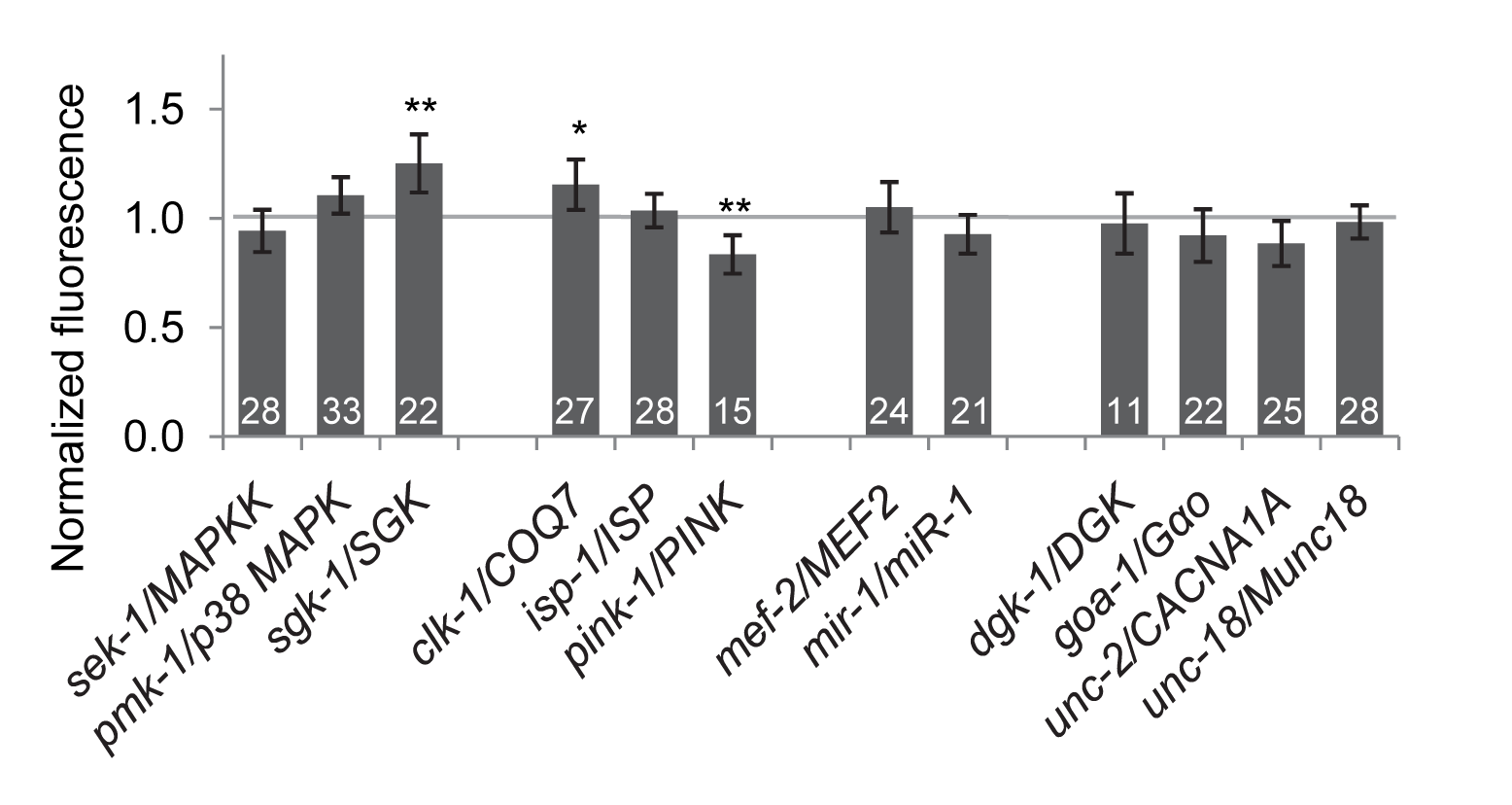

Supplement: Figure S3 — Quantification of Pnlg-1::gfp (vjIs47) in the ventral cord of animals in different genetic backgrounds. Samples sizes shown. *p<0.05, **p<0.01. (TIF) [file pgen.1004100.s003.tif]

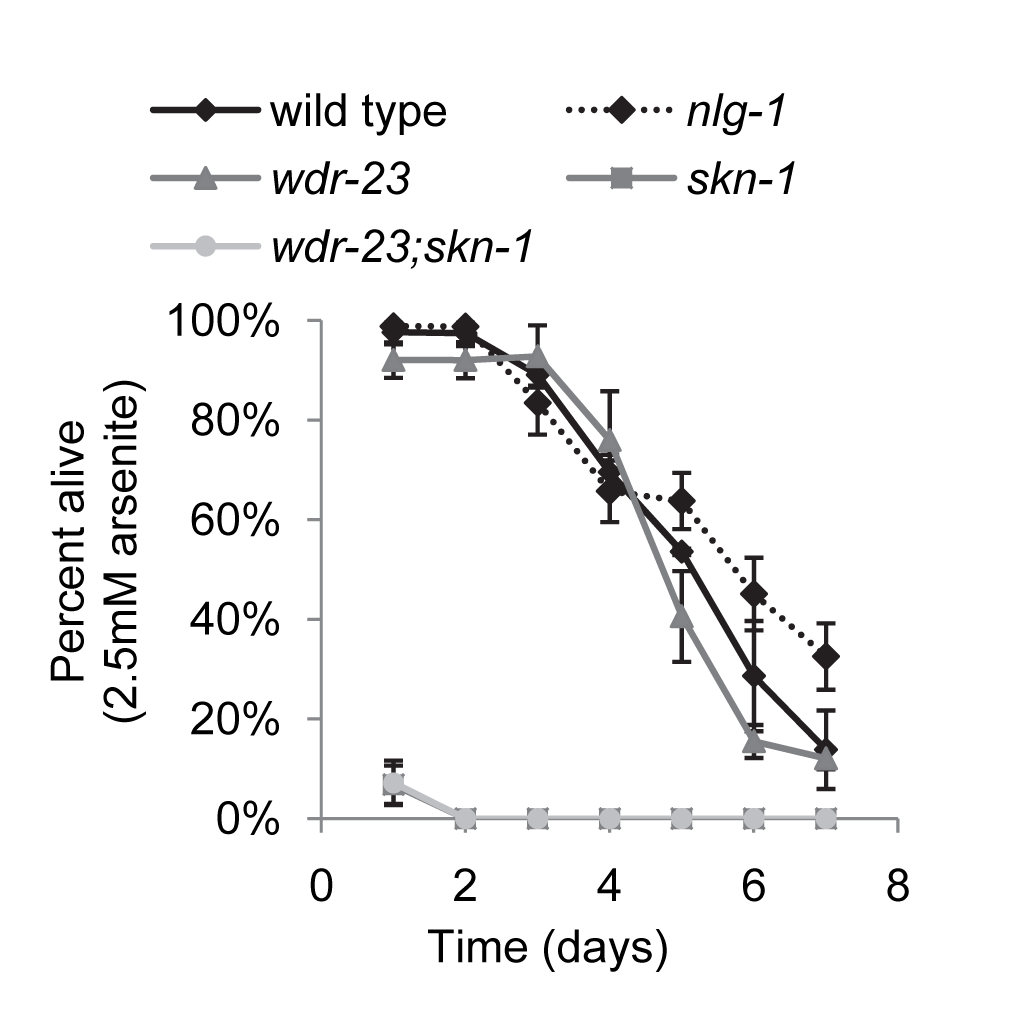

Supplement: Figure S4 — Survival in response to sodium arsenite. Survival curves of indicated strains on 2.5 mM sodium arsenite. Error bars represent ±sem. (TIF) [file pgen.1004100.s004.tif]

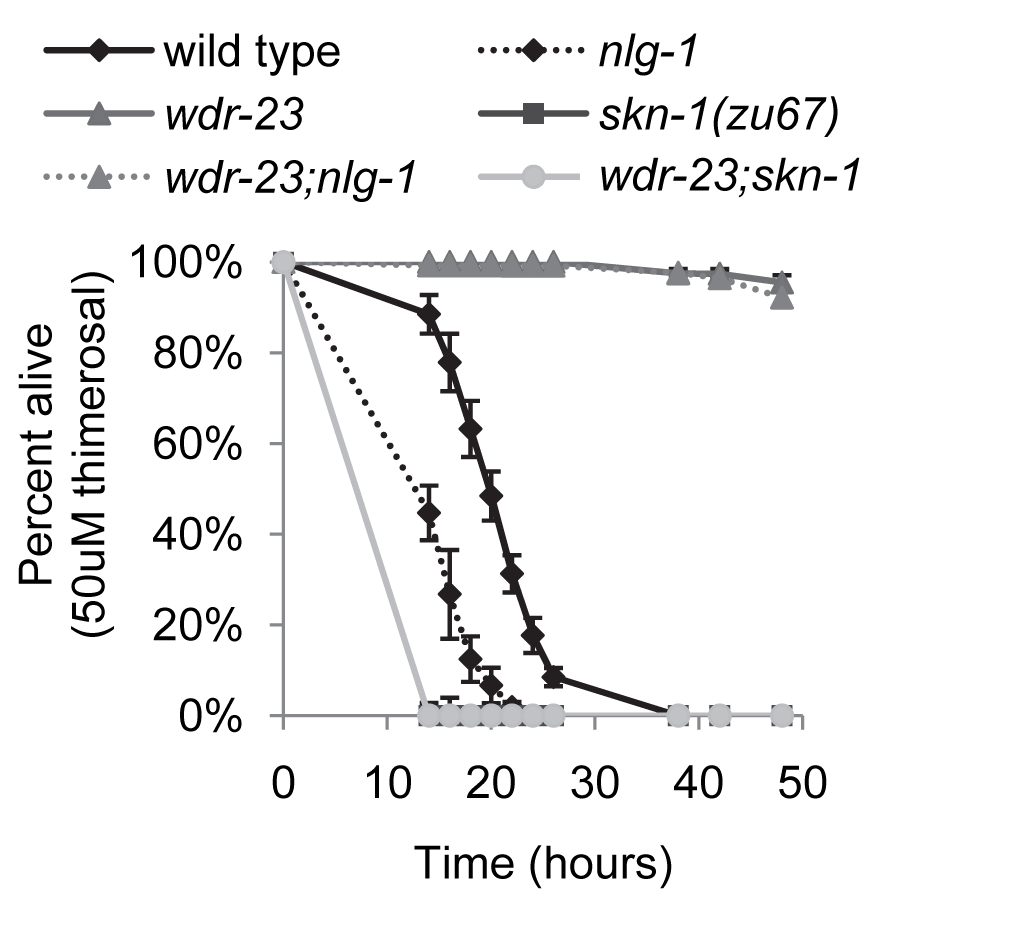

Supplement: Figure S5 — Survival in response to thimerosal. Survival curves of indicated strains on 50 µM thimerosal. Error bars represent ±sem. (TIF) [file pgen.1004100.s005.tif]

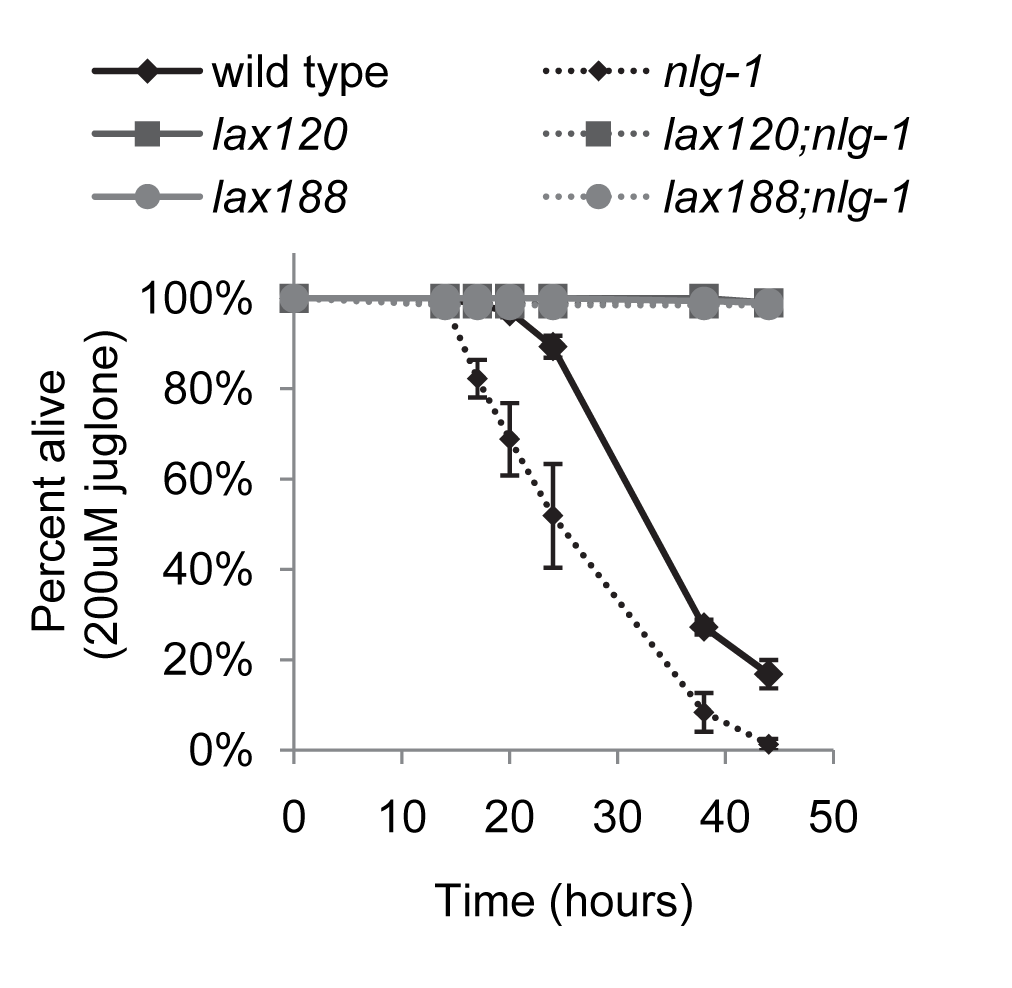

Supplement: Figure S6 — Survival of skn-1(gf) on 200 µM juglone. Survival curves of indicated strains on 200 µM juglone. Error bars represent ±sem. (TIF) [file pgen.1004100.s006.tif]

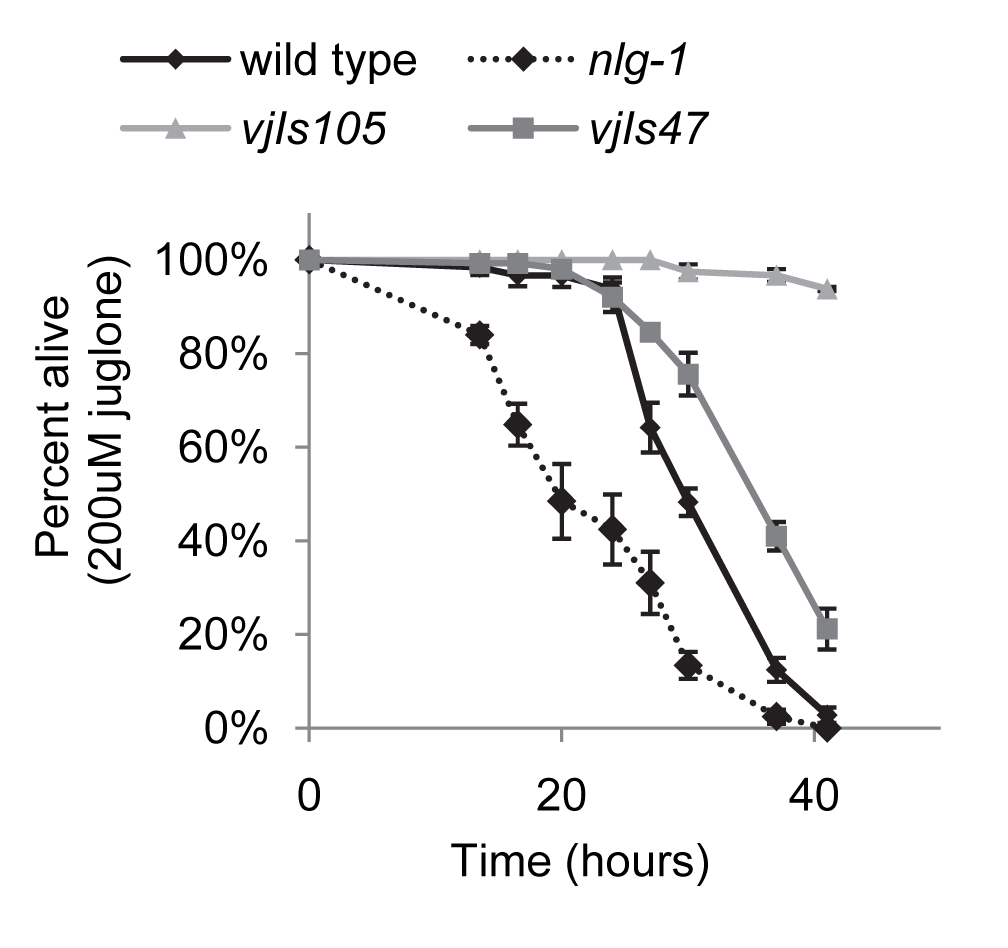

Supplement: Figure S7 — Survival of Pnlg-1::gfp on 200 µM juglone. Survival curves of indicated strains on 200 µM juglone. Error bars represent ±sem. (TIF) [file pgen.1004100.s007.tif]
