# Supplementary material for: Regulation of Synaptic nlg-1/Neuroligin Abundance by the skn-1/Nrf Stress Response Pathway Protects against Oxidative Stress
Source: PLoS Genet. 2014 Jan 16;10(1):e1004100. doi: 10.1371/journal.pgen.1004100 (PMC3894169; doi:10.1371/journal.pgen.1004100)
Supplement: Table S2 — Punctal analysis of NLG-1-GFP fluorescence. Raw values ± sem reported for peak, cord, width, and interpunctal interval (IPI). (PDF) [file pgen.1004100.s009.pdf]

Table S2. Punctal analysis of NLG-1-GFP fluorescence.

	Peak (arb. units)	Cord (arb. units)	Width (µm)	IPI (µm)
**Figure 5**				
**Dorsal**				
vjIs105, n=31	42.63±2.25	6.72±0.91	0.72±0.02	3.68±0.12
vjIs105;lax188, n=29	56.16±2.51***	10.43±1.44	0.84±0.05*	3.47±0.20
**Ventral**				
vjIs105, n=32	467.08±20.77	159.89±11.49	1.34±0.04	3.28±0.10
vjIs105;lax188, n=26	567.43±18.14***	214.56±10.53***	1.49±0.05*	3.32±0.08
**Figure 6C and 6D**				
vjIs105 DMSO, n=30	38.13±1.82	3.84±0.64	0.72±0.16	3.53±0.10
vjIs105 120uM juglone, n=29	50.49±1.88***	5.26±0.91	0.74±0.02	3.36±0.11

*$p<0.05$, **$p<0.01$, ***$p<0.001$
