# Supplementary material for: Regulation of Synaptic nlg-1/Neuroligin Abundance by the skn-1/Nrf Stress Response Pathway Protects against Oxidative Stress
Source: PLoS Genet. 2014 Jan 16;10(1):e1004100. doi: 10.1371/journal.pgen.1004100 (PMC3894169; doi:10.1371/journal.pgen.1004100)
Supplement: Table S3 — Statistical analysis of toxicity assays. Samples sizes, χ2 values for log rank tests, and significant differences between samples reported. Statistically significant differences were calculated using the log rank test with a Bonferroni correction to establish the thresholds of significance. (PDF) [file pgen.1004100.s010.pdf]

Table S3. Statistical analyses of toxicity assays.

	N	Comparison	Chi Sqr	dF	Threshold of significance
**Figure 7A**					
N2	116	N2 vs nlg-1*	32.38	4	0.005
nlg-1	68	N2 vs skn-1*	200.50		
wdr-23	114	N2 vs skn-1:wdr-23*	208.57		
skn-1	128	N2 vs wdr-23*	149.02		
skn-1;wdr-23	131	skn-1 vs skn-1;wdr-23	0.01		
**Figure 7B**					
N2	157	N2 vs nlg-1	3.53	6	0.002
wdr-23	79	N2 vs wdr-23*	203.33		
nlg-1	170	N2 vs lax120*	217.61		
lax120	108	N2 vs lax188*	184.92		
lax188	92	lax120 vs lax120;nlg-1*	58.31		
lax120;nlg-1	141	lax188 vs lax188;nlg-1*	61.72		
lax188;nlg-1	89				
**Figure 7C**					
N2	96	N2 vs nlg-1*	85.54	5	0.003
nlg-1	75	N2 vs vjIs105*	217.54		
vjIs105;nlg-1	137	N2 vs vjIs105;nlg-1*	90.45		
vjIs105;nlg-1	81	N2 vs vjEx561*	62.68		
vjEx561	104	N2 vs vjEx561;nlg-1	0.00		
vjEx561;nlg-1	68	nlg-1 vs vjIs105*	259.03		
		nlg-1 vs vjIs105;nlg-1*	141.00		
		nlg-1 vs vjEx561*	172.67		
		nlg-1 vs vjEx561;nlg-1*	46.15		
		vjIs105 vs vjIs105;nlg-1*	16.67		
		vjEx561 vs vjEx561;nlg-1*	38.99		
**Figure S4**					
N2	62	N2 vs nlg-1	3.12	4	0.005
nlg-1	64	N2 vs wdr-23	2.32		
wdr-23	71	N2 vs skn-1*	117.91		
skn-1	53	N2 vs wdr-23;skn-1*	99.72		
wdr-23;skn-1	76	skn-1 vs wdr-23;skn-1	0.06		

Table S3. Cont.

	N	Comparison	Chi Sqr	dF	Threshold of significance
**Figure S5**					
N2	161	N2 vs nlg-1*	99.16	5	0.003
nlg-1	108	N2 vs wdr-23*	358.51		
wdr-23	156	N2 vs skn-1*	213.20		
skn-1	120	N2 vs wdr-23;skn-1*	213.20		
wdr-23;nlg-1	113	N2 vs wdr-23;nlg-1*	271.61		
wdr-23;skn-1	120	skn-1 vs wdr-23;skn-1	0.33		
		wdr-23 vs wdr-23;nlg-1	1.39		
**Figure S6**					
N2	133	N2 vs nlg-1*	37.92	5	0.003
nlg-1	82	N2 vs lax120*	170.66		
lax120	113	N2 vs lax188*	180.68		
lax120;nlg-1	73	lax120 vs lax120;nlg-1	0.10		
lax188	125	lax188 vs lax188;nlg-1	0.00		
lax188;nlg-1	64				
**Figure S7**					
N2	95	N2 vs nlg-1*	42.01	3	0.008
nlg-1	89	N2 vs vjIs47*	29.14		
vjIs47	112	N2 vs vjIs105*	228.82		
vjIs105	132	vjIs47 vs vjIs105*	145.50		

**p* value statistically less than the Bonferroni-corrected threshold of significance.
